# Enhancement of Optical Transparency and Electrical Conductivity of IZO/Ag/IZO Multilayer Film by Intense Pulsed Light and its Effect on the Photovoltaic Performances of Perovskite Solar Cells

**DOI:** 10.1002/advs.202501058

**Published:** 2025-04-15

**Authors:** Sumin Bae, Youngsoo Jung, Vishal Pal, Jung‐Kun Lee

**Affiliations:** ^1^ Department of Mechanical Engineering & Material Science University of Pittsburgh Pittsburgh PA 15261 USA

**Keywords:** flash lamp annealing, optoelectronics, solar cells, thin metal films, transparent electrodes

## Abstract

A highly conductive and transparent oxide/metal/oxide (OMO) multilayer transparent electrode is developed by flash lamp annealing (FLA). A transient thermal effect of FLA on the sandwich structure of the ultrathin Ag layer and zinc‐doped indium oxide (IZO) layer is systematically investigated. FLA enables IZO/Ag/IZO multilayer to maintain the continuous ultrathin Ag interlayer and improve the crystallinity of the IZO layers. This is due to a very short processing time, absorption of visible light by the Ag layer, heat transfer from the Ag layer to the IZO layer, and mechanical constraint of the Ag layer by neighbor IZO layers. This combination of continuous ultrathin Ag layer and highly crystalline IZO layer decreases light scattering in a visible range and allows the electron donation from the Ag layer of a high electron concentration to neighbor IZO layers of a high electron mobility. IZO/Ag/IZO multilayer film from an optimal FLA process achieves a very low sheet resistance of 4.1 Ω sq^−1^ and a high optical transmittance (90.1%) in the broadband range of 400–800 nm. A perovskite solar cell in the best IZO/Ag/IZO transparent electrode exhibits better current generation and higher fill factor than a device of FTO electrode.

## Graphical abstract

The highly conductive IZO/Ag/IZO multilayer transparent electrode is successfully fabricated through flashlight annealing (FLA) process. A transient thermal effect of FLA maintains the continuous Ag interlayer and improves the crystallinity of the sandwich IZO layers. This combination increases both electric conductivity and optical transmittance. A perovskite solar cell in the best IZO/Ag/IZO transparent electrode exhibits better current generation and higher fill factor than a device of FTO electrode.

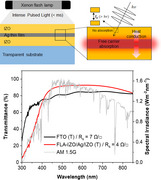

## 1 Introduction

A transparent electrode is an important component of various optoelectronic devices such as photovoltaics (PVs), light‐emitting diodes (LEDs), and touch panels.^[^ ^1^, ^2^, ^3^ ^]^ Recently, many studies have been conducted on transparent electrodes based on oxide/metal/oxide (OMO) multilayer structures.^[^ ^4^, ^5^, ^6^, ^7^, ^8^, ^9^, ^10^, ^11^ ^]^ In these OMO structures, high transparency can be achieved by placing two anti‐reflection oxide layers on the top and bottom of the metal film. However, there has been a trade‐off between electrical conductivity and optical transparency which are determined by the morphology and thickness of thin metallic films. Most metals (Ag, Au, Cu, etc.) have high surface energy and strong interaction between adjacent atoms, so the film growth of metals starts with the Volmer‐Weber mode. A percolation threshold (i.e., a critical thickness to form a continuous film) for high electric conductivity tends to be thick (>10 nm), which reduces optical transparency.^[^ ^12^, ^13^ ^]^ Although various oxide layers^[^ ^6^, ^14^, ^15^, ^16^, ^17^, ^18^, ^19^ ^]^ have been tested to improve the wetting of the metal film in the OMO structure, complete wetting does not occur in most cases because the adhesion energy of the metal‐oxide interface is lower than the surface energy of the metal film. To resolve this problem, the 1–2 nm thick adhesion layer of active metals (e.g., Ti, Cr) has been added on top of the oxide layer.^[^ ^20^, ^21^, ^22^, ^23^, ^24^, ^25^, ^26^ ^]^ The morphology of the metal ultrathin films on the 1–2 nm thick metal layer coated oxides is more continuous and uniform since the overall surface energy of the substrate is increased and sufficient nucleation sites for metal adatoms are available. However, the use of metal wetting layers can cause negative effects on both electrical and optical properties. Common wetting metals, such as Cr, Ti, and Ni, show a large optical loss in the wavelength of visible and NIR regions. These metals may reduce or damp plasmonic resonances in various devices enhanced by positive plasmonic resonance effects.^[^ ^27^, ^28^ ^]^ Moreover, diffusion and segregation of the metals into the main electrode metal film often occur. This has a negative effect on optical transparency and electrical conductivity by forming undesired metal alloys or segregated metal defects that act as scattering or parasitic absorption sites.^[^ ^29^, ^30^ ^]^

Several studies report the enhancement of electrical conductivity and optical transparency through post‐annealing of the OMO structure.^[^ ^31^, ^32^, ^33^, ^34^ ^]^ However, the process window of post‐annealing for improved properties is narrow. Above a certain temperature, the lateral diffusion of metal atoms still leads to the formation of discontinuous islands even in a sandwich structure, resulting in a significant decrease in electrical conductivity.^[^ ^4^ ^]^ The use of adhesion such as C^r[^ ^32^ ^]^ can push up the annealing temperature limit, but it may introduce optical loss or segregation issues as mentioned above. Furthermore, the traditional thermal annealing that employs convection or conduction is unsuitable for application to flexible plastic substrates with limited thermal budgets due to their sluggish ramp‐up rates. This results in undesired heating of vulnerable substrates.^[^ ^35^ ^]^

Flash lamp annealing (FLA) is a transient thermal processing using intense pulsed light (IPL) from a xenon flash lamp.^[^ ^36^ ^]^ This photonic process transfers radiative heat from the lamp to the surface of the desired object which absorbs incident light. The light absorption and the heat release take a very short time (in the range from 100 µs to 100 ms). The transient aspect of the FLA process allows for efficient and rapid curing of thin films without damaging the substrates that are fragile at high temperatures.

In this work, we investigate the enhancement of optical transparency and electrical conductivity of an OMO multilayer electrode composed of a metallic Ag thin layer and dielectric zinc‐doped indium oxide (IZO) layers by the FLA process. The Ag layer absorbs intense and ultra‐short pulses and is heated up to 620 °C where the crystallinity of Ag and neighboring IZO layers are significantly increased. In addition, the short pulse time suppresses the formation of discontinuous Ag islands, even in the absence of a wetting layer. Ag has been chosen as the metallic layer because of its low absorption and high transmittance in a visible range compared to other metallic materials such as Au and Cu.^[^ ^37^ ^]^ IZO has been chosen as the antireflection layer of OMO due to its good carrier mobility. IZO is electrically conductive even in the amorphous state because its cations, In and Zn, have an electron configuration of 4d^10^5s^0^ and 3d^10^4s^0^. These vacant s‐orbitals lead to a non‐directional spherical spread of free electrons, which is needed to increase the mobility of amorphous films.^[^ ^38^ ^]^ The FLA of IZO/Ag/IZO multilayer transparent electrode achieves a very low sheet resistance of 4.1 Ω/□ and high optical transmittance of 92.3% at 550 nm (FoM = 111 × 10^−3^ Ω^−1^) and average 90.1% in the range of 400–800 nm (FoM = 87.1 × 10^−3^ Ω^−1^). This electrode was also tested as a front electrode of perovskite solar cells (PSCs). PSCs of the IZO/Ag/IZO electrode exhibit better current generation and higher fill factor than that of the FTO electrode.

## 2 Results

### 2.1 Design of IZO/Ag/IZO Multilayer Film for Flash Lamp Annealing

**Figure 1:**
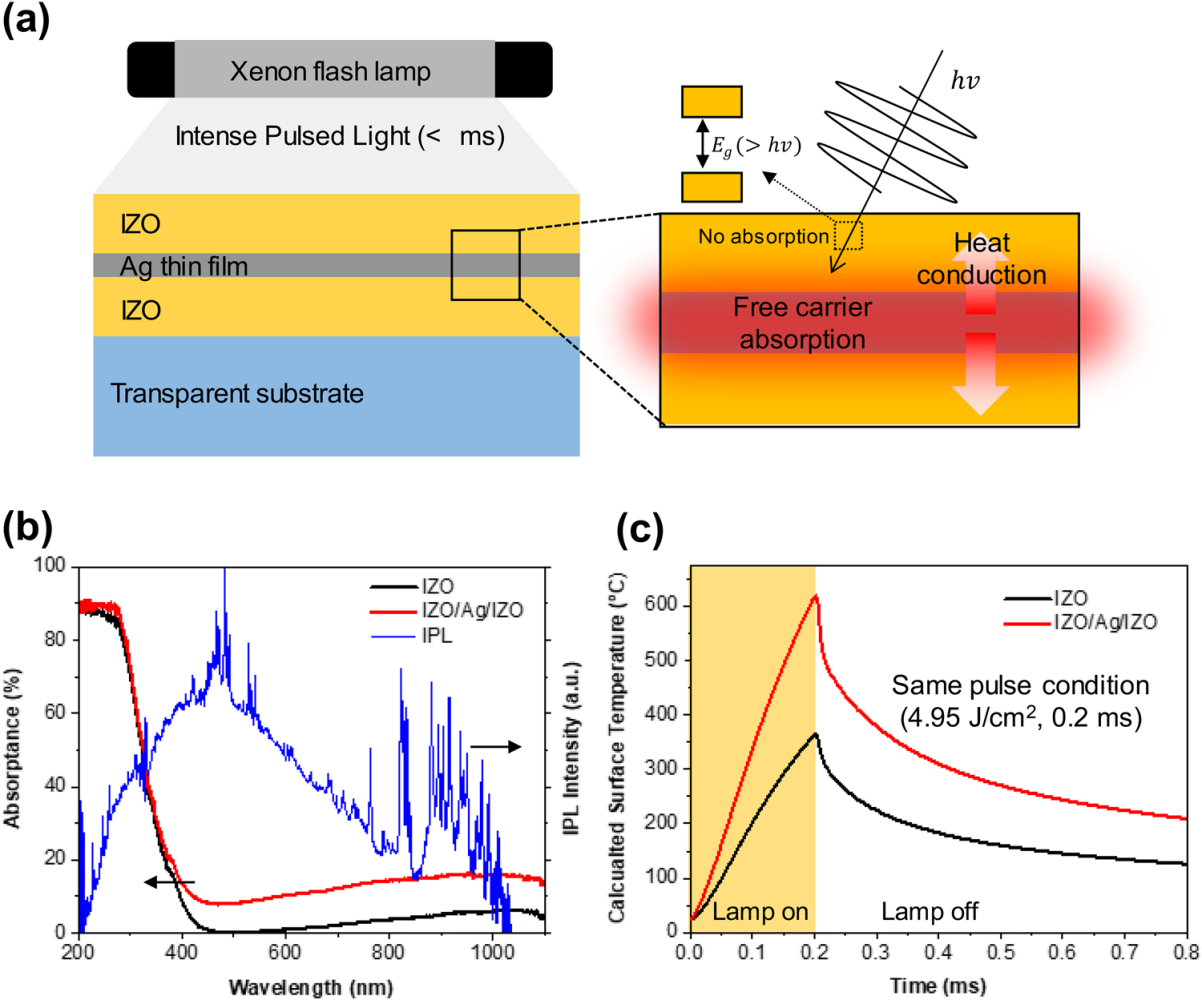
a) Schematic of the flash lamp annealing of IZO, Ag, and IZO/Ag/IZO using intense pulsed light (IPL), b) Absorptance of IZO and IZO/Ag/IZO and emission spectrum of IPL, c) Simulated surface temperature of IZO, Ag, and IZO/Ag/IZO under the same pulse condition (4.95 J cm^−2^, 0.2 ms).

The FLA process of IZO/Ag/IZO (40/8/40 nm) multilayer film is schematically explained in **Figure** **1**a. Although a wide band gap IZO (≈3.8 eV) is transparent except for UV light,^[^ ^39^ ^]^ the incorporation of the Ag interlayer can increase light absorption in the visible range due to light–free electron interaction. Unlike previously reported studies using conventional furnace annealing, this FLA using intense pulsed light has a very short annealing time so the Ag agglomeration at high temperatures by a long annealing process can be mitigated. Figure 1b shows the absorptance of IZO and IZO/Ag/IZO, and the radiant intensity spectrum of IPL generated from a xenon flash lamp. Since IZO is transparent for visible light, pure IZO film absorbs only the UV component of incident light. The insertion of the Ag layer between IZO layers makes the IZL/Ag/IZO multilayer film absorb a part of visible and IR light. This, in turn, increases the temperature of the multilayer film to be higher than that of pure IZO film by 250 °C. As shown in Figure 1c, the ultrathin Ag interlayer efficiently absorbs the radiant energy and heats the IZO/Ag/IZO multilayer stack at 620 °C for less than 0.2 ms.

The thickness of the Ag interlayer was fixed at 8 nm, which is the threshold thickness for forming a continuous layer in our experiments as shown in Figure S1 (Supporting Information). Island growth dominates at low deposition rates, leading to reduced electrical conductivity and plasmonic absorption. As the deposition rate increases, a more continuous layer is formed by suppressing surface‐diffusion‐enabled agglomeration.^[^ ^11^ ^]^ However, excessively high deposition rates make it challenging to control the thickness. At a thickness below 7 nm, Ag film shows relatively low conductivity and transparency. At the optimal deposition rate (5 Å/s), 8 nm thick Ag film is a continuous layer with high conductivity and transparency. The optical response of the multilayer film was calculated using a Finite‐Difference Time Domain (FDTD) simulation. The thickness of the Ag layer was fixed at 8 nm and the thickness of IZO varied from 0 to 100 nm. The multiple reflections at the interfaces and the absorption of each layer were considered in the simulation. The simulation result points out that the average transmittance in the wavelength range of 400–800 nm is maximized when the thickness of both the bottom and top IZO layers is ≈40 nm (see Figure S2, Supporting Information).

It is known that very thin and rough films with a few nanometers thickness exhibit higher light absorption than thick and smooth films. This is because the Volmer‐Weber growth mode of very thin films makes the surface rough and increases the light absorption in visible and NIR regions via multiple scatterings.^[^ ^40^, ^41^ ^]^ Figure S3 (Supporting Information) shows the normalized electric field distribution in IZO/Ag/IZO multilayers with flat and rough Ag interlayers. The morphology of the rough Ag interlayer was simulated based on the surface roughness information obtained from AFM analysis of an actual Ag thin film deposited on an IZO/Glass substrate, which is shown in Figure S4 (Supporting Information). In the case of the flat surface, the electric field is weak in the Ag layer, and the light absorption by the intermediate Ag layer is negligible.

The electric resistivity of IZO, Ag, and IZO/Ag/IZO layers in an as‐deposited state is shown in Table S1 (Supporting Information). Note that the measured resistivity (5.96 × 10^−5 ^Ω cm) of the IZO/Ag/IZO sample is much lower than the calculated one (7.89 × 10^−5 ^Ω cm) using the parallel mixture rule of the resistivity value of the IZO layer and the Ag layer. This implies that the IZO/Ag/IZO multilayer is not simply a parallel resistor consisting of IZO and Ag and that carrier donation at the interface may occur to improve the electric conductivity.^[^ ^42^, ^43^ ^]^ Ag has a work function value of 4.2–4.4 eV^[^ ^42^, ^44^ ^]^ and IZO has a work function value of 4.4–5.1 eV.^[^ ^45^, ^46^, ^47^ ^]^ Hence, an ohmic contact is formed at the interface between the Ag and IZO, and free electrons of the Ag layer can be transferred to the IZO layer without energy cost.^[^ ^42^, ^43^ ^]^ By combining the high carrier concentration of Ag and the high carrier mobility of IZO, IZO/Ag/IZO can theoretically exhibit high conductivity (Table S1, Supporting Information) due to the donation of free electrons from Ag to IZO.

### 2.2 Enhancement of Optical Transparency and Electrical Conductivity of the Multilayer Film by Intense Pulsed Light

**Figure 2:**
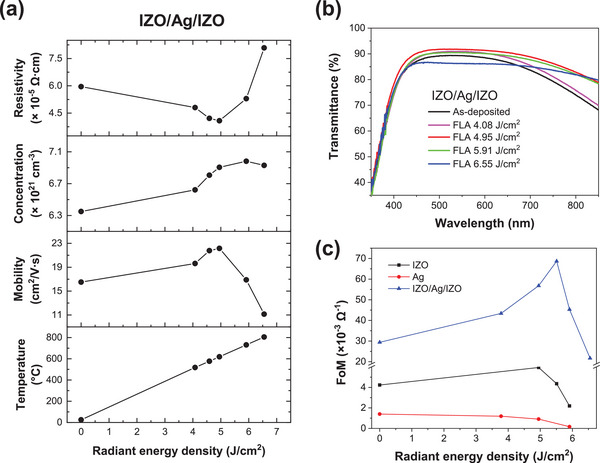
a) Electrical properties (resistivity, carrier concentration, and mobility) and estimated peak temperature of IZO/Ag/IZO multilayer films as a function of radiant energy density, and b) corresponding optical transmittance. c) Haacke's figure of merit (FoM) of IZO, Ag, and IZO/Ag/IZO as a function of radiant energy density at 550 nm.

**Figure** **2**a shows the electrical properties of IZO/Ag/IZO multilayer films after the FLA process. A change in the radiant energy has a clear effect. The FLA process decreases the resistivity of IZO/Ag/IZO from 5.9×10^−5^ down to 4.0×10^−5^ Ω⋅cm (i.e., a decrease in the sheet resistance from 6.77 to 4.64 Ω/□) and increases the optical transparency in the visible range (400 < λ < 800 nm) from 85.1% to up to 89.2%. **Table** **1** presents the electrical and optical properties of IZO, Ag, and IZO/Ag/IZO films under different FLA conditions. It is noted that the FLA process improves the figure of merit (FoM) of the IZO/Ag/IZO film by 134% (29.4 × 10^−3^ Ω^−1^ → 68.7 × 10^−3^ Ω^−1^), due to 32% decrease in sheet resistance and 4.8% increase in transmittance (85.1% → 89.2%). In this study, the FoM value was calculated using Haacke's method^[^ ^48^ ^]^ defined by φ_H_ = T^10^/R_s_, where T is the average transmittance of 400–800 nm wavelength range and R_s_ is the sheet resistance. For the specific wavelength 550 nm, the FoM value is 90.6 × 10^−3^ Ω^−1^. The FoM values of the flash lamp annealed IZO/Ag/IZO multilayer films are much higher than those of the IZO and Ag single‐layer films (Figure 2c). The effect of FLA is explained by the light absorption and heat generation of the embedded Ag layer. Ag in the IZO/Ag/IZO multilayer absorbs radiant energy, heats neighbor IZO layers, and heals the internal defects of IZO. This, in turn, increases the electron mobility and optical transparency of IZO.

**Table 1:** Electrical and optical properties, and Haacke's figure of merit (FoM) of IZO, Ag, and IZO/Ag/IZO as a function of radiant energy density with the fixed pulse duration of 0.2 ms.

Materials	Radiant Energy Density [J/cm^2^]	Sheet Resistance [Ω/□]	Carrier Concentration [× 10^21^ cm^−3^]	Carrier Mobility [cm^2^/V∙s]	T_400–800_ _nm_ [%]	FoM [× 10^−3^ Ω^−1^]
IZO (88 nm)	0	46.5	0.444	34.4	85	4.23
	4.95	37.7	0.501	37.6	86.1	5.94
	5.51	49.0	0.379	44.2	85.7	4.36
	5.91	95.3	0.119	62.8	85.5	2.19
	6.55	‐	‐	‐	84.4	0
Ag (8 nm)	0	10.9	53.2	13.5	65.8	1.40
	3.78	9.55	54.1	15.1	63.9	1.19
	4.95	10.2	53.6	14.3	62.6	0.91
	5.91	30.8	48.7	5.21	59	0.17
	6.55	‐	‐	‐	54.9	0
IZO/Ag/IZO (40/8/40 nm)	0	6.77	6.35	16.5	85.1	29.4
	4.08	5.46	6.62	19.6	86.6	43.5
	4.59	4.79	6.81	21.8	87.8	56.8
	4.95	4.64	6.91	22.2	89.2	68.7
	5.91	6.01	6.98	16.9	87.8	45.3
	6.55	9.19	6.93	11.1	85.1	21.7

The effect of FLA of individual IZO or Ag single layer on the properties is presented in Figure S5 (Supporting Information). An increase in the radiant energy from 1 to 5 J cm^−2^ slightly decreases the resistivity of the IZO single layer. Further increasing radiant energy (up to 7 J cm^−2^) dramatically increases the resistivity of the IZO single layer. This result is consistent with previous studies reporting changes in the electrical properties of IZO induced by conventional thermal annealing.^[^ ^39^, ^49^, ^50^ ^]^ As the radiant energy increases to 6 J cm^−2^, the IZO film is quickly heated to 430 °C where the primary electron sources such as oxygen vacancies or Zn interstitials are almost removed through their reaction with oxygen in air.^[^ ^49^, ^50^ ^]^ X‐ray photoelectron spectroscopy (XPS) data in Figure S6 (Supporting Information) confirm that oxygen vacancies are reduced by FLA. The results show that IPL has an impact on the defect states of oxygen. There are two O1s peaks of IZO. The IPL annealing decreases the relative intensity of the O1s peak at 532 eV, which has been assigned as an oxygen vacancy or other oxygen‐related defects such as adsorbed oxygen or OH‐. The XPS data suggests that the IPL annealing reduces the oxygen‐related defects and increases the crystallinity of the IZO layer. Such a decrease in a concentration of point defects, in turn, decreases a free electron concentration (Figure S5a, Supporting Information). A shift of a transmittance edge toward a longer wavelength by higher radiation energy is evidence of a reduced electron concentration. This is known as the Moss‐Burstein effect of degenerate semiconductors.^[^ ^39^ ^]^ At much higher radiant energy (6.55 J cm^−2^), the IZO electrode loses its electric conductivity, because the extreme thermal gradient between the IZO and glass substrate promotes the crack formation (Figure S7, Supporting Information).

In the Ag single layer, the increase in the radiant energy to 5 J cm^−2^ does not change the resistivity of the film. Further increase in the radiation energy to 6.55 J cm^−2^ increases the electric resistivity rapidly. The FLA process of Ag films has two competing factors: the reduction of grain boundary scattering by the growth of Ag grains and the discontinuation of Ag films by the island formation. The former increases the conductivity, while the latter decreases it. Figure S8 (Supporting Information) shows AFM images of the Ag single layer after the FLA process. As the radiation energy increases to 6.55 J cm^−2^, isolated islands appear. This self‐organization phenomenon of thin Ag film is consistent with previous reports on the thermal annealing effect.^[^ ^51^, ^52^, ^53^ ^]^ As the radiant energy increases, the transmittance of the Ag single layer decreases at 300 < λ < 700 nm, while the transmittance above 700 nm increases (Figure S5d, Supporting Information). A transmittance valley near λ = 500 nm is due to localized plasmonic absorption caused by Ag nanoparticles. The peak position of the transmittance valley matches well with the peak wavelength of scattering cross‐section spectra of Ag nanoparticles (size: 50, 100, and 150 nm). Simulation results of Mie scattering of Ag nanoparticles are shown in Figure S9 (Supporting Information).

As a control sample, the IZO/Ag/IZO multilayer film is thermally annealed at 500 °C to compare the FLA annealing and the traditional furnace heating. The transmittance spectrum of thermally annealed IZO/Ag/IZO multilayer film in Figure S10 (Supporting Information) shows a hump at 500 nm. This suggests that the thermal annealing forms Ag‐isolated islands, though the IZO layers hold the Ag layer on both sides. The continuous Ag layer and the isolated Ag islands in the IZO/Ag/IZO film exhibit very different optical responses. In the case of the continuous layer, the transmitted and reflective lights at different interfaces cause destructive or constructive interference. As simulated in Figure S9 (Supporting Information), the plasmonic effect of Ag‐isolated islands causes an absorption peak and increases light scattering, thereby decreasing the transmittance in the visible region. Unlike the Ag single layer and the thermally treated IZO/Ag/IZO film, a plasmonic absorption peak is not observed in the FLA‐processed IZO/Ag/IZO multilayer films (Figure 2b). This suggests that the Ag interlayer maintains the continuous and smooth morphology of the intermediate Ag layer if the radiation energy is smaller than 6 J cm^−2^. It is important to point out that an increase in the radiation energy increases both the carrier concentration and carrier mobility of IZO/Ag/IZO multilayer film in Table 1. To explain the reduced resistivity of the IZO/Ag/IZO multilayer films by the FLA process, we need to consider the electron donation from the Ag layer to the IZO layer. Our previous study demonstrated that the Fermi energy level difference (work function of IZO > work function of Ag) formed the ohmic contact at the Ag‐IZO interface and caused the donation of free electrons from the Ag layer to the IZO layer.^[^ ^42^ ^]^ Figure S10 (Supporting Information) schematically explains band bending at an interface between Ag and IZO and the electron accumulation region near the IZO‐Ag interface. Because a decrease in the electron concentration by the FLA process lowers the Fermi level of the IZO, FLA facilitates such an electron donation from the Ag layer to the IZO layer. Given that the mobility of the IZO layer is higher than that of the Ag layer, free electrons donated by the Ag layer travel in the IZO layer of the high‐carrier mobility. Hence, the FLA process increases the density of free electrons which transport electricity through the high mobility layer (i.e., IZO) of the IZO/Ag/IZO film, which leads to a concomitant increase in the carrier concentration and carrier mobility in the FLA treated IZO/Ag/IZO multilayer film.

### 2.3 Crystal Structure and Microstructure of IZO/Ag/IZO Multilayer Film

**Figure 3:**
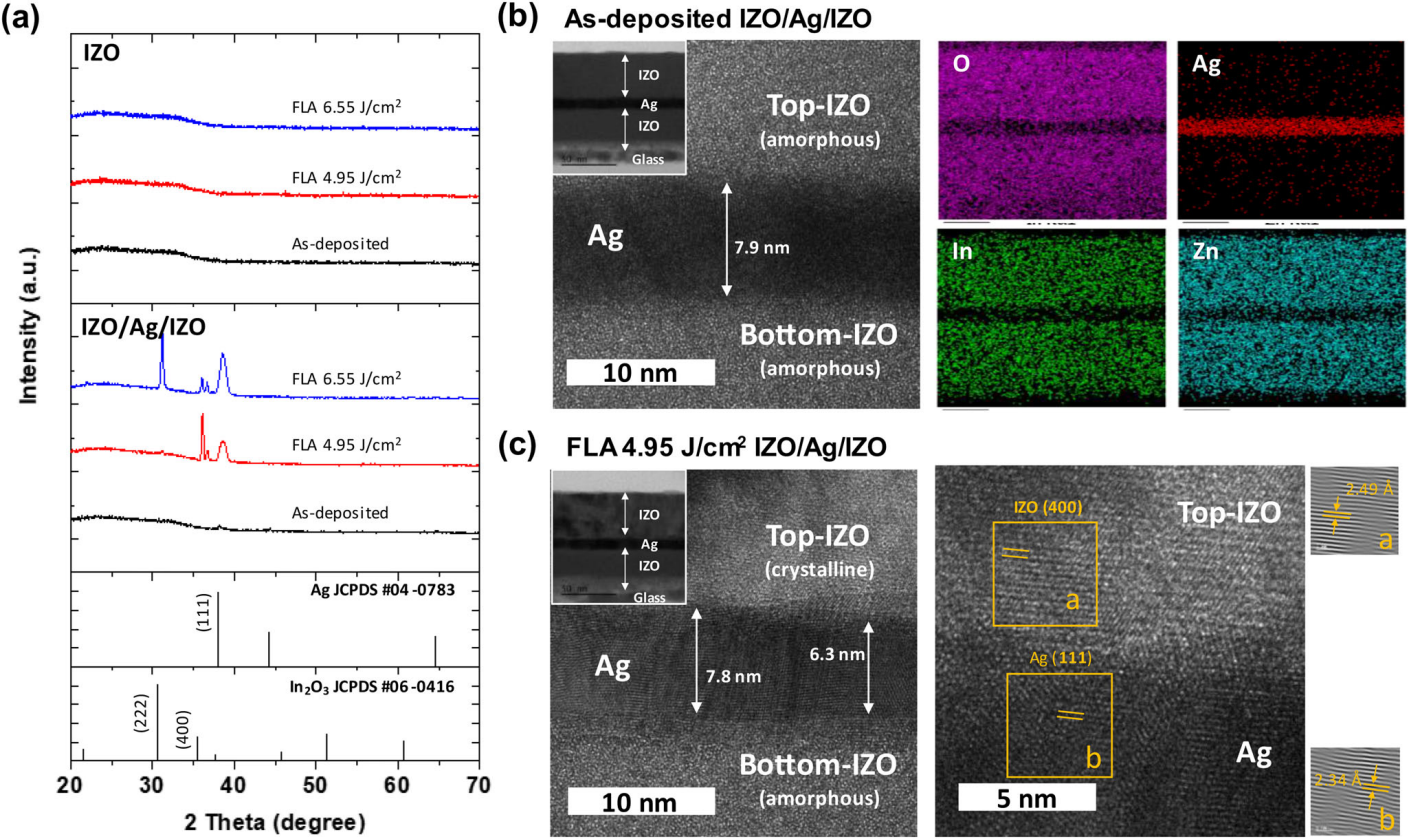
a) XRD spectra of IZO and IZO/Ag/IZO films under the same flash lamp annealing conditions. b) Cross‐sectional HR‐TEM image of as‐deposited IZO/Ag/IZO multilayer film and EDX elemental mapping of O, Ag, In, Zn. c) Cross‐sectional HR‐TEM image of flash lamp annealed IZO/Ag/IZO multilayer film and enlarged image at the interface between crystalline top‐IZO and Ag interlayer with Fourier filtered images of IZO (400) and Ag (111) planes.

To investigate the structural characteristics of IZO/Ag/IZO multilayer film, X‐ray diffraction (XRD) and high‐resolution transmission electron microscopy (HR‐TEM) were used. **Figure** **3**a shows XRD spectra of IZO and IZO/Ag/IZO films as‐deposited and after the FLA process. In the pure IZO film, the amorphous nature of the IZO layer does not change because pure IZO film absorbs only a UV portion of incident light. In contrast, the XRD pattern indicates that the IZO layer in IZO/Ag/IZO film is well crystallized at or above the radiant energy of 4.95 J cm^−2^. After flash lamp annealing at 4.95 J cm^−2^, strong XRD peaks of Ag (111) and IZO (400) are observed. This suggests that the insertion of the Ag interlayer between IZO layers effectively enhances the absorption of the incident light in a visible range and increases the temperature of the IZO layer, as schematically explained in Figure 1c. It is noted that the XRD pattern of highly crystalline IZO film is similar to that of body‐centered cubic In_2_O_3_ film.^[^ ^54^, ^55^ ^]^ In general, the thermodynamically stable (222) plane is the preferred growth orientation for doped In_2_O_3_ in an oxygen‐rich atmosphere while (400) plane can be preferentially oriented in an oxygen‐poor atmosphere.^[^ ^56^, ^57^ ^]^ At flash lamp annealing at 4.95 J cm^−2^, the oxygen‐deficient IZO (400) plane might appear, because the short annealing time suppresses the oxygen diffusion toward the IZO film and the IZO film is deficient in the oxygen content. At a much higher radiant energy 6.55 J cm^−2^, higher film temperature increases the oxygen diffusion toward IZO film and decreases the oxygen deficiency of the film. This change in the oxygen content of the film may cause a change in the dominant orientation of the IZO layer from (400) to (222).

Figure 3b shows cross‐sectional TEM images and elemental mapping of as‐deposited IZO/Ag/IZO film. There is a continuous 8 nm thick Ag interlayer between the top and bottom IZO layers. In the as‐deposited state, both IZO and Ag layers are amorphous, which is consistent with XRD results. Figure 3c shows cross‐sectional TEM images of IZO/Ag/IZO film illuminated with pulsed light of 4.95 J cm^−2^ and Fourier‐filtered images of the top IZO and center Ag layers. The d‐spacing of each plane corresponds to that of crystalline IZO (400) and Ag (111) planes. This also supports XRD results. It is worth noting that the top IZO layer is crystallized first at the energy of 4.95 J cm^−2^. This is attributed to the heat dissipation toward the glass substrate that is in contact with the bottom IZO layer. Such thermal conduction decreases the temperature of the bottom IZO layer faster than that of the top AZO layer. Calculated temperature profiles are shown in Figure S11 (Supporting Information).

### 2.4 Morphology Evolution of IZO/Ag/IZO Multilayer Film

**Figure 4:**
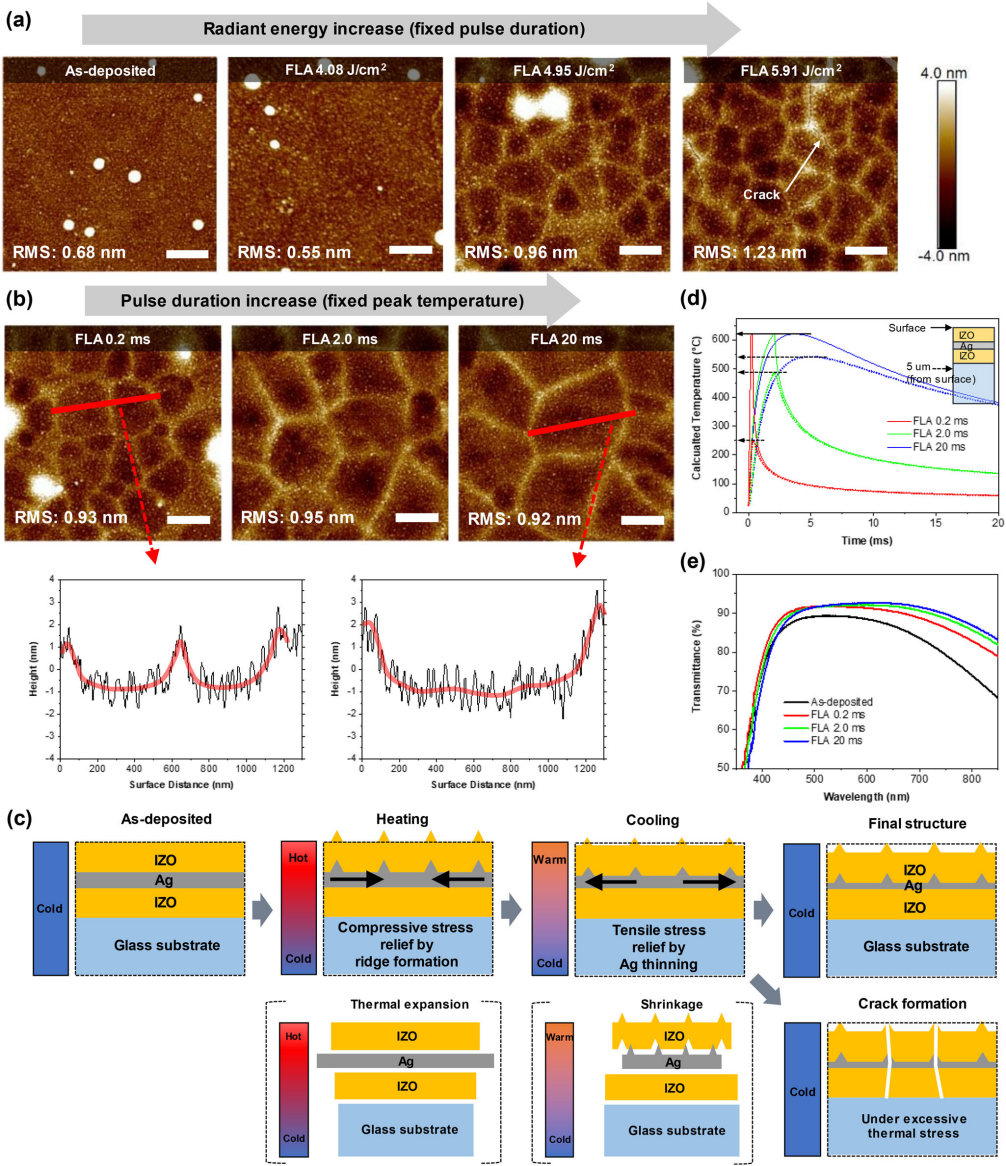
AFM images for surface morphology evolution of the IZO/Ag/IZO multilayer film depending on a) different radiant energy (4.08, 4.95, and 5.91 J cm^−2^) with the fixed pulse duration time (0.2 ms) and b) different pulse duration time (0.2, 2, and 20 ms) with the same peak temperature of 620 °C (the length of a scale bar is 500 nm). c) Schematic illustration of structural evolution of the IZO/Ag/IZO multilayer film during the flash lamp annealing process. d) A time‐dependent temperature profile of the center of the multilayer film (solid line) and the location of 5 µm below the center of the film (dotted line) and e) transmittance spectra of the IZO/Ag/IZO multilayer film depending on the pulse duration.

**Figure** **4**a shows a change in the surface morphology of the IZO/Ag/IZO multilayer film by atomic forced microscopy (AFM). All samples were exposed to different radiation energy with the same exposure time. The as‐deposited IZO/Ag/IZO film exhibits a granular surface feature composed of small grains with an average diameter of ≈20 nm. At the radiant energy 4.08 J cm^−2^, the surface roughness decreases from 0.68 to 0.55 nm and the average grain size increases to ≈31 nm. At the radiant energy 4.95 J cm^−2^ where the crystallization of IZO occurs, a surface undulation with characteristic polygonal features is observed. At the relatively high radiant energy 5.91 J cm^−2^, the distance between the polygonal boundaries decreases from 500 to 300 nm and tiny cracks are found. A similar crack is found in a previous study of IZO/Ag/AZO in which the Ag interlayer retards the crack evolution in the IZO layer.^[^ ^58^ ^]^ To better understand the morphology change, the annealing time at the peak temperature is changed. To keep the peak temperature at 620 °C, both radiation energy (4.95, 17.7, and 42.2 J cm^2^) and exposure time (0.2, 2.0, and 20 ms) are controlled. In this experiment, longer annealing decreases the temperature difference between the center of the multilayer and the depth of 5 µm below the multilayer from 370 °C (0.2 ms) to 70 °C (20 ms), as shown in Figure 4d). This lowers the thermal stress between the IZO/Ag/IZO layer and changes the undulation behavior. Figure 4b shows the AFM images of optically cured IZO/Ag/IZO films with a pulse duration of 0.2, 2.0, and 20 ms. Longer annealing and a decrease in the temperature gradient along the film thickness direction increase the lateral size of the polygonal features from 500 nm to 1 µm. The vertical edge height of the polygonal features also increases from 2 to 3 nm, which results from the surface undulation. Interestingly, fine cracks are not observed in the multilayer films after the 20 ms long FLA. The increase in the size of the undulated polygonal feature and the disappearance of fine cracks improve the optical transmittance (Figure 4e).

A proposed structural evolution and crack formation of the IZO/Ag/IZO multilayer film during the FLA process is illustrated in Figure 4c. When the IZO/Ag/IZO film on the glass substrate is quickly heated under the pulsed light, the Ag interlayer during the heating experiences a strong in‐plane compressive stress because of a large difference in thermal expansion between Ag and IZO (α_*Ag*_ = 18–20 × 10^−6^ /K, α_*IZO*_ = 3–8 × 10^−6^ /K). This thermal stress can be relieved by the form of hillocks.^[^ ^59^, ^60^ ^]^ Since the FLA process limits the diffusion of Ag atoms, the Ag layer keeps an interconnected structure and a wrinkle‐like ridge surface appears rather than a few large islands to relax the compressive stress. As the stress‐relaxed multilayer with a ridged surface cools down, a difference in the thermal expansion inversely generates in‐plane tensile stress in the Ag layer. This causes the partial dewetting and thinning of the Ag layer.^[^ ^61^ ^]^

**Table** **2** shows the electrical and optical properties of IZO/Ag/IZO multilayer film which is annealed at the same peak temperature (620 °C) with different pulse duration times. With an increase in the pulse duration time, the average transmittance from 400 to 800 nm slightly increases. Notably, the increase in the pulse duration time enhances the conductivity by increasing the carrier mobility. This is because the longer pulse duration promotes the crystallization of the IZO layer where free electrons travel. The 0.2 ms duration crystallizes only the top IZO layer, while the 20 ms duration crystallizes both the top and bottom IZO layers (Figure S12, Supporting Information). However, due to a decrease in oxygen vacancies in IZO layers, the overall carrier concentration saturates at a certain level. The champion flash lamp annealed IZO/Ag/IZO electrode shows the highest FoM value of 111 × 10^−3^ Ω^−1^, which is much higher than that of different oxide/metal/oxide transparent electrodes reported so far (Table S3, Supporting Information).

**Table 2:** Electrical and optical properties of IZO/Ag/IZO multilayer film which are annealed at the same peak temperature with different pulse duration times.

Pulse Duration [ms]	Radiant Energy Density [J/cm^2^]	Resistivity [× 10^−5^ Ω∙cm]	Sheet Resistance [Ω/□]	Carrier Concentration [× 10^21^ cm^−3^]	Mobility [cm^2^/V∙s]	Average Transmittance [%, 400–800 nm]
‐	‐	5.96	6.77	6.35	16.5	85.1
0.2	4.95	4.08	4.64	6.91	22.2	89.1
2.0	17.7	3.73	4.24	6.83	24.5	89.9
20	42.2	3.56	4.05	6.80	25.8	90.1

**Figure 5:**
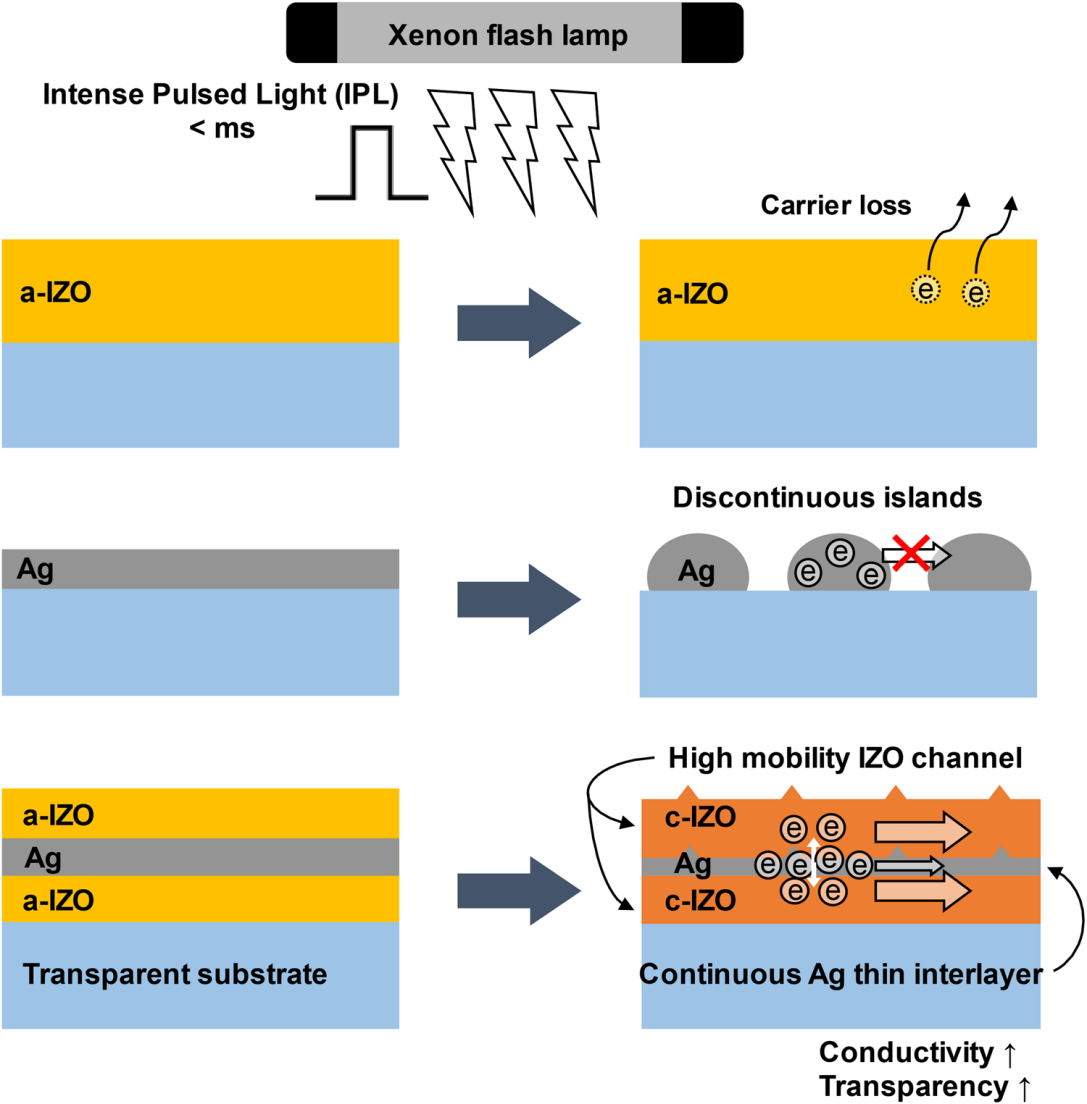
Schematic illustration of flash lamp annealing process for IZO single layer, Ag single layer, and IZO/Ag/IZO multilayer electrodes.

**Figure** **5** schematically shows the electron transport in the IZO single layer, Ag single layer, and IZO/Ag/IZO multilayers after the FLA process. For the IZO single layer, the FLA process decreases the electron concentration and increases the carrier mobility by increasing the crystallinity of the IZO layer. For the Ag single layer, the FLA process increases the grain size but causes the dewetting of the Ag layer and the formation of discontinuous islands. In the case of the IZO/Ag/IZO multilayer, these drawbacks of the IZO and Ag layers are resolved. The IZO/Ag/IZO multilayer efficiently absorbs the incident flashlight and increases the crystallinity of the IZO layer without the dewetting issue of the Ag layer. This, in turn, improves the mobility and concentration of free electrons and the optical transmittance.

### 2.5 IZO/Ag/IZO Multilayer as the Transparent Electrode of Perovskite Solar Cells

**Figure 6:**
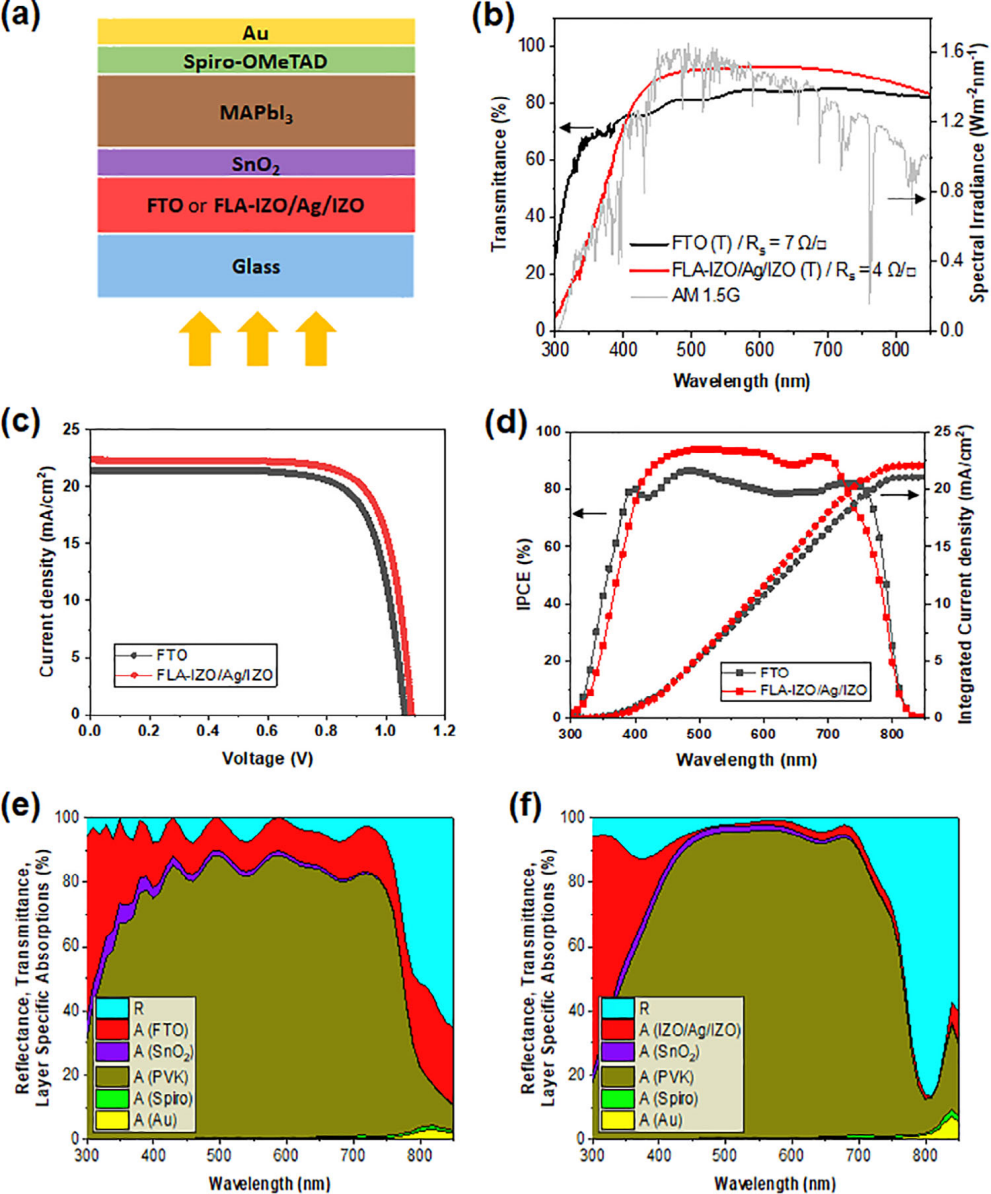
a) Schematic cross‐section of IZO/Ag/IZO‐based perovskite solar cell device. b) Optical transmittance of FTO and IZO/Ag/IZO. c) J‐V curves and d) IPCE of FTO‐based and FLA‐IZO/Ag/IZO‐based solar cell devices. Optical response of e) FTO‐based and f) IZO/Ag/IZO‐based solar cell devices.

To demonstrate the feasibility of the FLA‐processed IZO/Ag/IZO film as the transparent electrode, MAPbI_3_‐based perovskite solar cells were fabricated on the flash lamp annealed IZO/Ag/IZO (FLA‐IZO/Ag/IZO) coated glass. The device structure is shown in **Figure** **6**a. The photovoltaic performance of the devices with the FLA‐IZO/Ag/IZO was compared with the devices with fluorine‐doped tin oxide (FTO), which is widely used as a transparent electrode in perovskite solar cells. Figure 6b shows a comparison between commercially available FTO (TEC‐7) and FLA‐IZO/Ag/IZO with AM 1.5G illumination. While the FTO exhibits higher transmittance in the UV region, FLA‐IZO/Ag/IZO has higher transmittance in the visible region, where the spectral irradiance is much higher. Since most photocurrent generation in the actual MAPbI_3_ active layer primarily occurs in the visible region, the devices with the FLA‐IZO/Ag/IZO electrode are expected to have higher current density. Figure 6c and **Table** **3** present the photovoltaic performance of represented devices with the FTO and the FLA‐IZO/Ag/IZO. Compared to the FTO‐based device, the short‐circuit current density (J_SC_) and fill factor (FF) of the FLA‐IZO/Ag/IZO‐based device are increased from 21.0 to 22.4 mA cm^−2^ and 0.69 to 0.74, respectively. The higher optical transmittance of the FLA‐IZO/Ag/IZO reduces optical losses and improves photo‐current conversion. The lower sheet resistance of the FLA‐IZO/Ag/IZO electrode decreases the series resistance (R_s_) of the device from 4.71 to 2.98 Ω cm^−2^. Furthermore, the shunt resistance (R_sh_) of the FLA‐IZO/Ag/IZO‐based device increases from 1.09 to 6.98 kΩ cm^−2^. The lower R_sh_ is attributed to the fact that the surface roughness of the FLA‐IZO/Ag/IZO electrode (RMS ≈1 nm) is much lower than that of the TEC7‐FTO electrode (RMS ≈15 nm). Smoother surfaces reduce the chances of forming shunt paths, resulting in higher shunt resistance and the fill factor. The combined effect of low series resistance and high shunt resistance improves the overall fill factor of the FLA‐IZO/Ag/IZO‐based device. Furthermore, the open‐circuit voltage (V_OC_) of the FLA‐IZO/Ag/IZO‐based device is increased from 1.07 to 1.09 V. Although the enhancement of V_OC_ is marginal, this improvement is attributed to a different work function. Ultraviolet photoelectron spectroscopy (UPS) results in Figure S13 (Supporting Information) show that the work function of the FLA‐IZO/Ag/IZO (4.5 eV) is smaller than that of the FTO (4.8 eV) so Fermi energy levels are better aligned at the interface between FLA‐IZO/Ag/IZO and SnO_2_. Statistical data on J_SC_, V_OC_, FF, and PCE are shown in Figure S14 (Supporting Information). The statistical distribution suggests that the performance improvement by FLA‐IZO/Ag/IZO is mainly caused by the increase of J_SC_ and FF. It is worth noting that the V_OC_ distribution of the FLA‐IZO/Ag/IZO‐based devices is narrower than that of the FTO‐based devices indicating that a shunt‐driven V_OC_ loss which is closely related to a manufacturing defect is suppressed. As shown in Figure 6d, the integrated current densities of the FTO‐based and the FLA‐IZO/Ag/IZO‐based perovskite solar cells based on the incident photon to current efficiency (IPCE) are 20.4 and 22.0 mA cm^−2^, respectively, which show a good agreement with the J‐V curve. The amount of light absorbed by each layer constituting the devices was simulated as shown in Figure 6e,f. The FLA‐IZO/Ag/IZO front electrode decreases parasitic optical loss compared to the FTO front electrode. The results of Figure 6 suggest that the FLA of IZO/Ag/IZO transparent electrodes has the potential to enhance the photovoltaic performances of perovskite solar cell devices in comparison to TEC‐7 FTO transparent electrodes.

**Table 3:** Represented photovoltaic properties of FTO and IZO/Ag/IZO‐based perovskite solar cells.

Electrode	J_SC_ [mA/cm^2^]	V_OC_ [V]	FF [%]	PCE [%]	R_sh_ [kΩ·cm^2^]	R_s_ [Ω·cm^2^]
FTO	21.43	1.06	0.74	17.04	3.77	4.53
FLA‐IZO/Ag/IZO	22.37	1.09	0.76	18.44	6.92	2.86

## 3 Conclusion

In this study, the highly conductive IZO/Ag/IZO multilayer transparent electrode was successfully fabricated and the effect of FLA on the multilayer films was systematically investigated. The FLA process with controlled radiation energy and duration time effectively crystallizes the multilayer film and eliminates structural defects while maintaining the continuity of the Ag interlayer without cracking. By sandwiching an ultrathin Ag interlayer between the IZO layers, the advantages of the high carrier mobility of IZO and the high carrier concentration of Ag are synergistically combined. This leads to the IZO/Ag/IZO multilayer film of optical transparency and electrical conductivity. In addition, the FLA of IZO/Ag/IZO multilayer was successfully applied to the fabrication of the front transparent electrode of PSCs. In comparison to TEC‐7 FTO, the IZO/Ag/IZO layer increases J_SC_ and FF of the device. This shows the potential of the FLA process in manufacturing the multilayer transparent electrode for electric and optical devices.

## 4 Experimental Section

### IZO/Ag/IZO Multilayer Deposition

Ag (99.99%, Kurt J. Lesker) and IZO (90 wt.% In_2_O_3_ + 10 wt.% ZnO, 99.99%, iTASCO) targets were used for the sputtering system (NexDep, Angstrom). Prior to deposition, the glass substrates (1mm thick were cleaned with acetone, methanol, and de‐ionized (DI) water under ultrasonication. The ultrathin Ag film was deposited at a DC power of 60 W, an Ar flow rate of 15 sccm, and a working pressure of 3 mTorr. The deposition rate of Ag was calibrated to 5 Å/s. Note that thicknesses less than 8 nm show a significant absorption valley in the visible region due to the island formation that is observed below the percolation threshold. For the IZO deposition, an RF power of 66 W, an Ar flow rate of 15 sccm, and a working pressure of 3 mTorr was conducted. The deposition rate of IZO was calibrated to 1 Ȧ/s. For the DMD configuration, the Ag thickness was fixed at 8, and 40 nm of IZO was deposited on both sides of the Ag interlayer.

### Flash Lamp Annealing Process

As‐deposited films were photonically annealed using a commercial flash lamp annealing system (Pulseforge 1300, Novacentrix) under the ambient environment. The size of the irradiated beam was uniformly generated by 150 × 75 mm. The radiant energy density of a single pulse was controlled by the voltage of the lamp and the duration of the irradiation. The radiant exposure was measured using a National Institute of Standards and Technology (NIST)‐traceable bolometer, and 1 J cm^−2^ of a single pulse was calibrated by controlling parameters. The distance between the lamp housing and the surface of the sample was set to 7 mm which is the same distance as used for calibration. The surface temperature of the sample was calculated by SimPulse software which simulates a provided material stack. The heat conduction of the material stack by pulse irradiation is modeled based on 1D heat conduction Equation (1),^[^ ^62^ ^]^

(1) $$\rho c_{P}\;\frac{\partial T}{\partial t}=\frac{\partial }{\partial x}\;\left(k\frac{\partial T}{\partial x}\right)$$

where *T* is the temperature, *t* is the time, *x* is the depth from the top surface, ρ is the density, *c_P_* is the specific heat at constant pressure, and *k* is the thermal conductivity. For the total thickness of the material L, when the heat pulse is treated as a surface flux, the boundary conditions at *x* = 0 (the top surface boundary) and *x* = L (the bottom surface boundary) are expressed as Equations (2) and (3),^[^ ^62^ ^]^

(2) $${q^{\prime \prime }}_{0}\;\left(t\right)=-h_{\infty ,0}\left(T_{0}-T_{\infty ,0}\right)-\varepsilon \sigma T_{0}^{4}$$

(3) $${q^{\prime \prime }}_{L}\;\left(t\right)=h_{\infty ,L}\;\left(T_{L}-T_{\infty ,L}\right)$$

where ${q^{\prime \prime }}_{x}\left(t\right)$ are the pulse waveform, σ is the Stefan‐Boltzmann constant, ε is the emissivity, *h*_∞,*x*_ is the convective heat transfer coefficient, *T*_0_ is the surface temperature, *T*_∞,0_ is the ambient surface temperature above the film stack. Thermal coefficients used for the SimPulse simulation are presented in Table S2 (Supporting Information).

To compare with the furnace heating, the samples were placed in a preheated furnace to the desired temperature and then removed immediately. The time taken for a sample to reach thermal equilibrium in the furnace was calculated by Newton's cooling law, Equation (4),

(4) $$-mc_{P}\;\frac{dT}{dt}=hA\left(T-T_{\infty }\right)$$

where *m* is the mass, *c_P_* is the specific heat at constant pressure, *h* is the heat transfer coefficient of alumina on which the sample was placed, *A* is the surface area in contact with the alumina plate, *T* is the initial temperature of the specimen, and *T*_∞_ is the desired temperature.

### Device Fabrication

The prepared electrodes, FTO (TEC‐7, Pilkington), and flash lamp annealed IZO/Ag/IZO, were cleaned with acetone, DI water, and ethanol in an ultrasonic bath for 15 min each. The cleaned substrates were treated with ultraviolet ozone (UV‐O) for 15 min. The aqueous SnO_2_ solution (15% in H_2_O colloidal dispersion, Alfa Aesar) was diluted in DI water with a ratio of 2.67 wt.%, and then it was spin‐coated on the UV‐O treated substrates at 3000 rom for 30 s, followed by annealing at 150 °C for 30 min. After the substrates were cooled down at room temperature, a perovskite layer (MAPbI_3_, CH_3_NH_3_PbI_3_) was deposited. The MAPbI_3_ solution was prepared by dissolving methylammonium and iodide (CH_3_NH_3_I) lead iodide (PbI_2_) with a molar ratio of 1:1 in the mixture of dimethyl sulfoxide (DMSO) and N,N‐dimethylformamide (DMF). The MAPbI_3_ solution was spin‐coated on top of the SnO_2_/electrode substrates at 4000 rpm for 25 s. During the spinning, 0.5 mL of diethyl ether (anti‐solvent) was dropped at 18 s on the MAPbI_3_ wet layer. Subsequently, the sample was annealed at 100 °C for 10 min. Then, 2‐phenylethylammonium iodide (PEAI) was spin‐coated to passivate the surface of the perovskite layer. A Spiro‐OMeTAD solution, which was prepared by mixing 72 mg mL^−1^ of Spiro‐OMeTAD in chlorobenzene with 28.8 uL of 4‐tert‐butylpyridine (tBP) and 17.6 uL of lithium bis(trifluoromethylsulphonyl)imide (Li‐TFSI) solution (720 mg mL^−1^ in acetonitrile) was spin‐coated on top of the perovskite layer at 4000 rpm for 30 s. Finally, an Au electrode thicker than 50 nm was thermally evaporated on top of the Spiro‐OMeTAD layer.

### Characterizations

The electrical properties of the fabricated samples were examined using a hall‐effect measurement system (HMS‐5000, Ecopia) at room temperature. Sheet resistance was measured by using the van der Pauw technique and mobility was calculated using the carrier concentration value obtained from the Hall measurement. The transmittance and reflectance of the samples were measured by an ultraviolet‐visible (UV–vis) spectrophotometer (Lambda 35, PerkinElmer) equipped with an integrating sphere. The surface morphology of the samples was characterized by the tapping mode of atomic force microscopy (Dimension Icon AFM, Bruker). High‐resolution current mapping was conducted to analyze the DMD films using PeakForce TUNA mode of the same AFM equipment. The crystal structure of the thin films was analyzed by an X‐ray diffraction system (D8 Discover XRD, Bruker). The chemical state and elemental analysis were performed using an X‐ray photoelectron spectrometer (ESCALAB 250Xi XPS, Thermo Fisher Scientific). The optical simulations were conducted based on the finite‐difference time‐domain (FDTD, Lumerical) method.

## Conflict of Interest

The authors declare no conflict of interest.

## Supporting information



Supporting Information

## Data Availability

The data that support the findings of this study are available from the corresponding author upon reasonable request.
