# Supplementary material for: Enhancement of Optical Transparency and Electrical Conductivity of IZO/Ag/IZO Multilayer Film by Intense Pulsed Light and its Effect on the Photovoltaic Performances of Perovskite Solar Cells
Source: Adv Sci (Weinh). 2025 Apr 15;12(25):2501058. doi: 10.1002/advs.202501058 (PMC12225018; doi:10.1002/advs.202501058)
Supplement: Supplementary file 1 — Supporting Information [file ADVS-12-2501058-s001.docx]

Supplementary Information

**Enhancement of Optical Transparency and Electrical Conductivity of IZO/Ag/IZO Multilayer using Intense Pulsed Light for Boosting Photovoltaic Performances of Perovskite Solar Cells**

Sumin Bae, Vishal Pal, Youngsoo Jung, and Jung-Kun Lee*

Department of Mechanical Engineering & Material Science, University of Pittsburgh, Pittsburgh PA 15261, USA

* Corresponding author: [jul37@pitt.edu](mailto:jul37@pitt.edu)

Supplementary Table 1. Electrical properties of as-deposited IZO, Ag and IZO/Ag/IZO films obtained by Hall effect measurement. The calculated resistivity of IZO/Ag/IZO is based on a parallel mixture rule.

| Materials | Thickness (nm) | Resistivity  ($\times$ 10^-5^ Ω∙cm) | Sheet resistance  (Ω/□) | Carrier mobility  (cm^2^/V∙s) | Carrier concentration  ($\times$ 10^21^ cm^-3^) |
| --- | --- | --- | --- | --- | --- |
| IZO | 88 | 40.9 | 46.5 | 34.4 | 0.444 |
| Ag | 8 | 0.87 | 10.9 | 13.5 | 53.2 |
| IZO/Ag/IZO | 40/8/40 | 5.96 | 6.77 | 16.5 | 6.35 |
| IZO/Ag/IZO  (Parallel mixture rule) | 40/8/40 | 7.89  (Calculation) | 8.97  (Calculation) | - | - |

Supplementary Table 2. Thermal coefficients used for the SimPulse simulation.

| Thermal coefficients | Mass density  (g/cm^3^) | Specific heat  (J/kg∙K) | Thermal conductivity  (W/m∙K) |
| --- | --- | --- | --- |
| Ag | 10.5 | 235 | 420 |
| IZO | 7.02 | 357 | 3.40 |
| Glass substrate | 2.52 | 800 | 0.80 |

Supplementary Table 3. Comparison of Haacke’s figure of merit (FoM) based on transmittance at 550 nm.

| Structure | Sheet resistance  (Ω/□) | T_550 nm_  (%) | FoM  (× 10^-3^ Ω^-1^) | Reference |
| --- | --- | --- | --- | --- |
| IZO/Ag/IZO  (as-deposited) | 6.77 | 89.3 | 47.6 | This work |
| IZO/Ag/IZO  (FLA 0.2 ms, 4.95 J/cm^2^) | 4.64 | 91.7 | 90.6 | This work |
| IZO/Ag/IZO  (FLA 20 ms, 42.2 J/cm^2^) | 4.05 | 92.3 | 111 | This work |
| AZO/Ag/AZO | 5.60 | 93.6 | 92.2 | ^8^ |
| TiO_2_/Ag/AZO | 5.75 | 91.6 | 72.3 | ^6^ |
| TiO_2_/Ag/ITO | 6.20 | 88.6 | 48.5 | ^5^ |
| ITO/Ag/ITO | 9.51 | 88.2 | 30.0 | ^4^ |
| Commercial FTO  (TEC-7) | 7.07 | 83.2 | 22.5 | - |


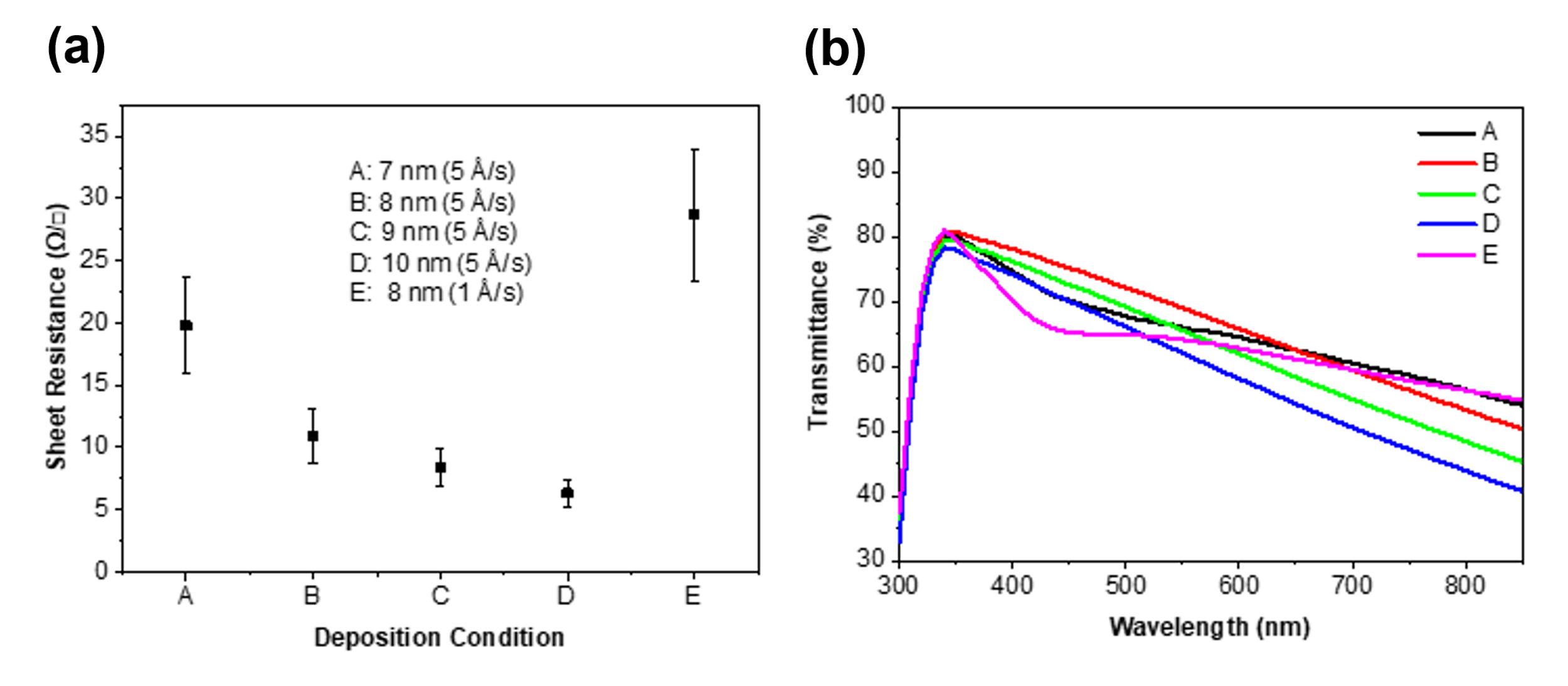


Supplementary Figure 1. (a) Sheet resistance and (b) transmittance of Ag thin film prepared under various deposition conditions (A: 7 nm – 5 Å/s, B: 8 nm – 5 Å/s, C: 9 nm – 5 Å/s, D: 10 nm – 5 Å/s, E: 8 nm – 1 Å/s). Island growth dominates at low deposition rates, leading to reduced electrical conductivity and plasmonic absorption. At thickness below 7 nm shows relatively low conductivity and transparency. At the optimal deposition rate (5 Å/s), the silver film formed a continuous layer with high conductivity and transparency when depositing a threshold thickness of 8 nm.


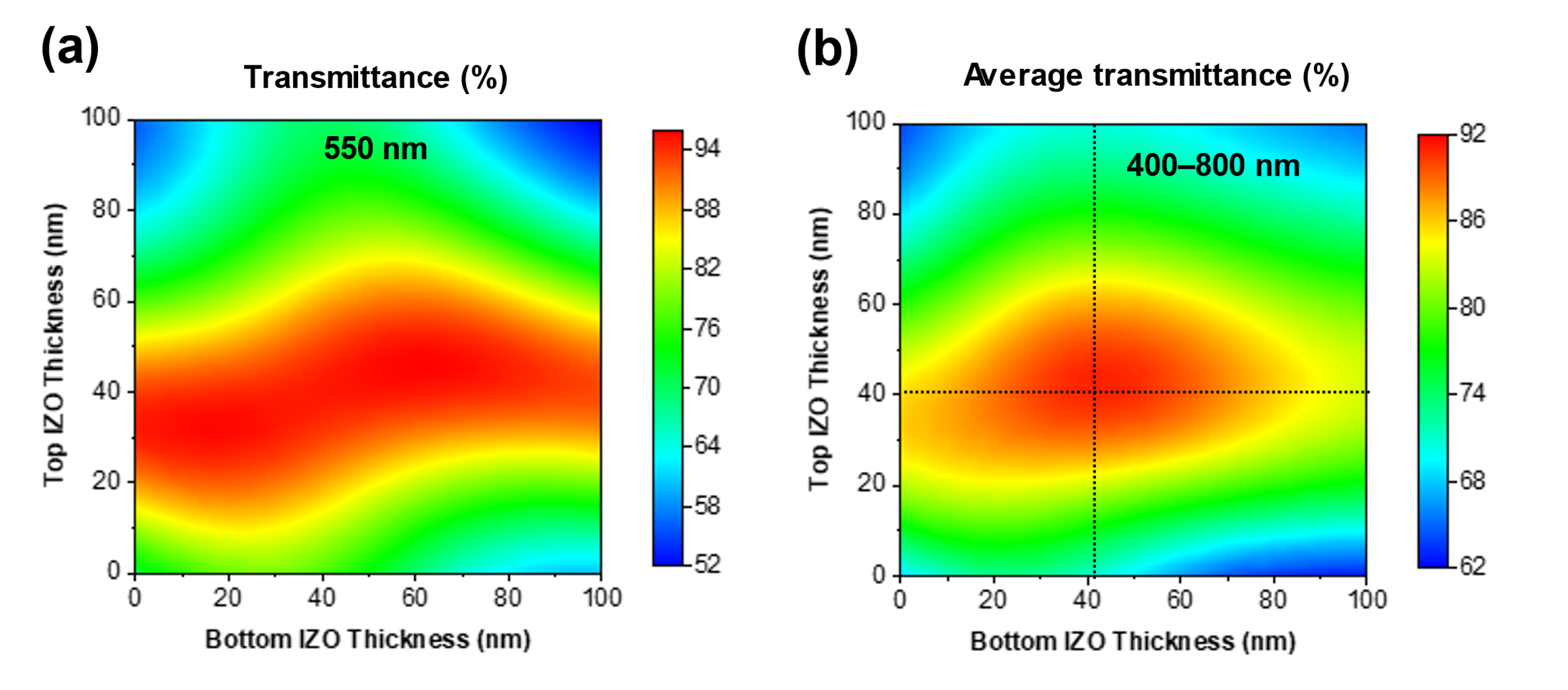


Supplementary Figure 2. Simulated (a) transmittance at 550 nm wavelength, and (b) average transmittance over 400-800 nm wavelength range of as-deposited IZO/Ag/IZO multilayer film with the fixed 8 nm thick Ag inter layer.


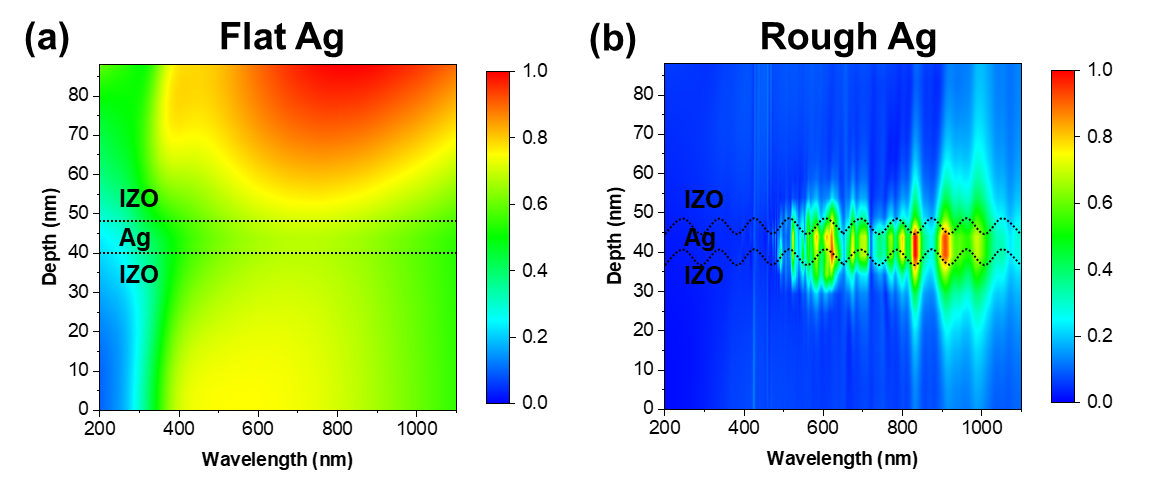


Supplementary Figure 3. Normalized electric field distribution of IZO/Ag/IZO (40/8/40 nm) multilayers with (a) flat and (b) rough Ag interlayers over 200-1100 nm wavelength range. The rough Ag interlayer is modeled based on a grain size of 25 nm and the root-mean-square (RMS) roughness of 2 nm. Note that the electric field of the rough Ag layer is stronger than that of the flat Ag layer.


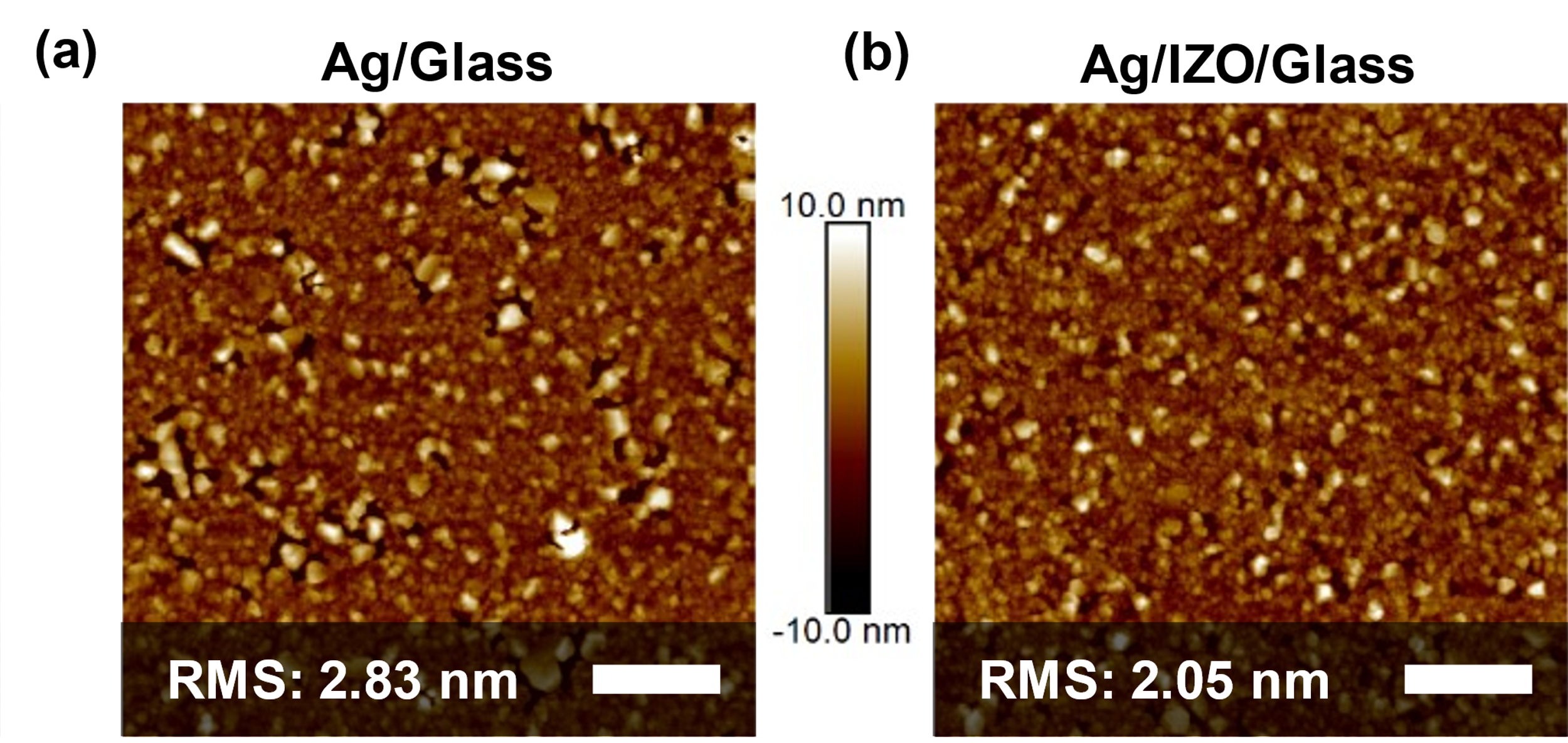


Supplementary Figure 4. (a) AFM images of Ag thin film deposited on (a) glass and (b) IZO/Glass. The scale bar is 500 nm.


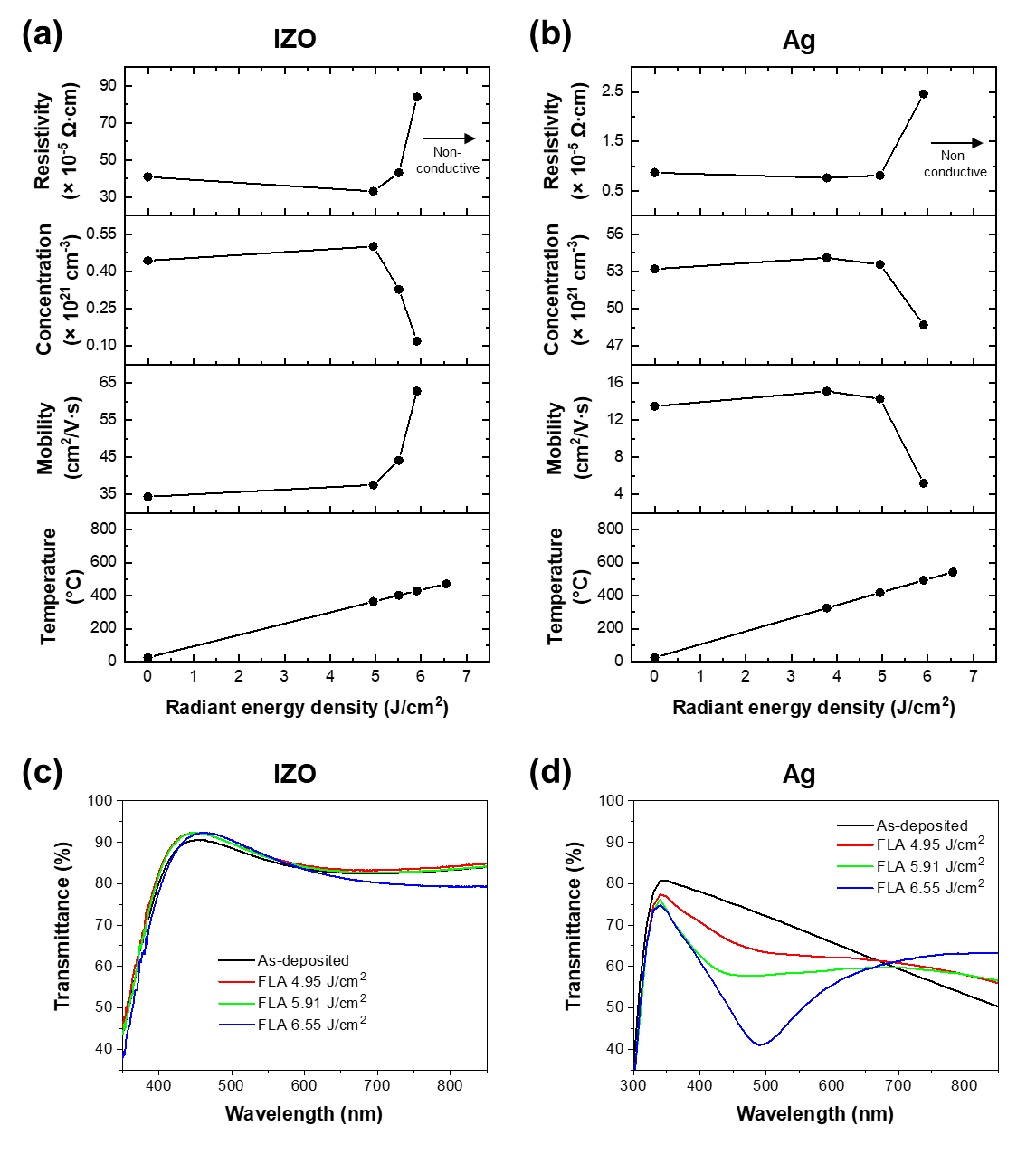


**Supplementary Figure 5.** Electrical properties (resistivity, carrier concentration, and mobility) and estimated peak temperature of (a) IZO (88 nm) and (b) Ag (8 nm) as a function of radiant energy density, and corresponding optical transmittance of (c) IZO and (d) Ag.


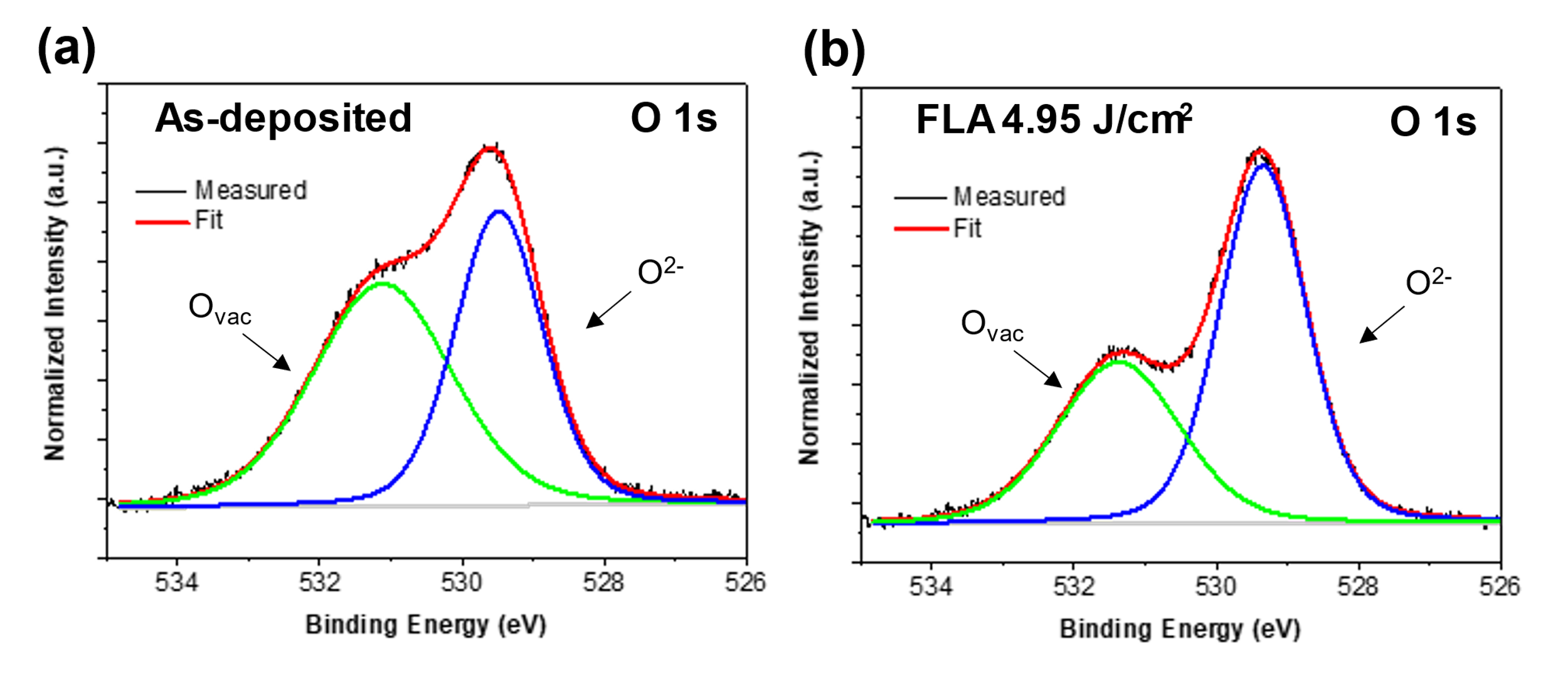


Supplementary Figure 6. X-ray photoelectron spectroscopy (XPS) of O1s for (a) as-deposited and (b) flash lamp annealed IZO/Ag/IZO multilayer film. The O 1s spectrum shows a combined peak which consists of two kinds of oxygen, the one at the lower binding energy 529.6 eV can be ascribed to the lattice oxygen (In-O-In or Zn-O-Zn), and the other one at the higher binding energy 531.5 eV can be ascribed to the vacant oxygen (oxygen defects).


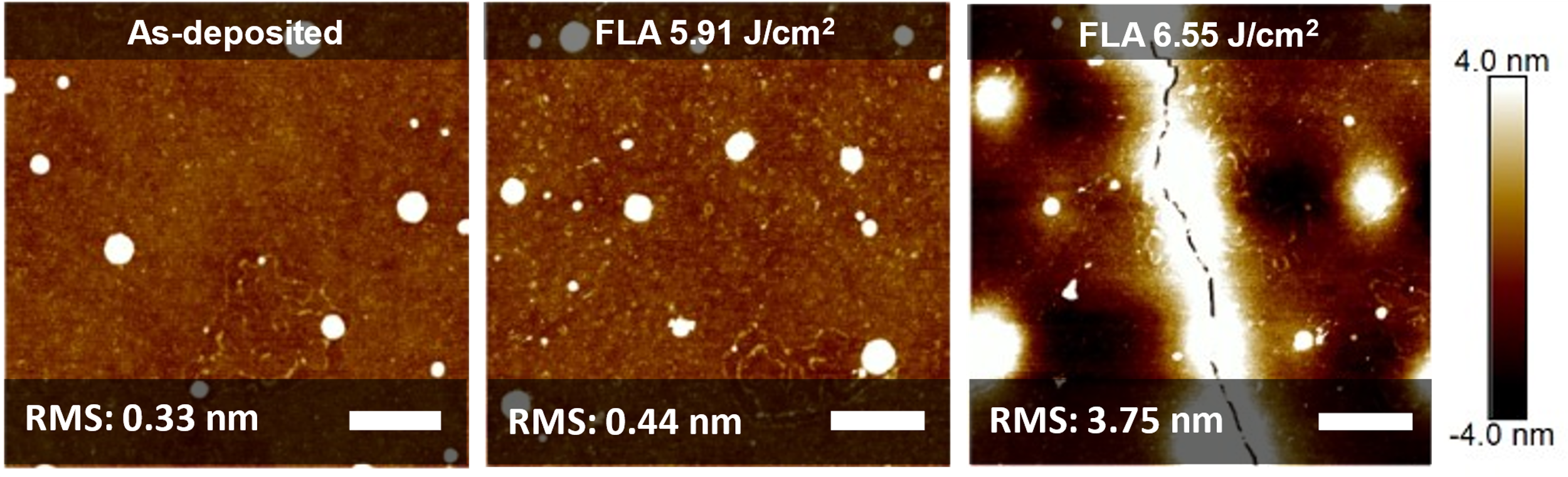


Supplementary Figure 7. Atomic force microscopy (AFM) images of as-deposited and flash lamp annealed IZO single layer films. The scale bar is 500 nm.


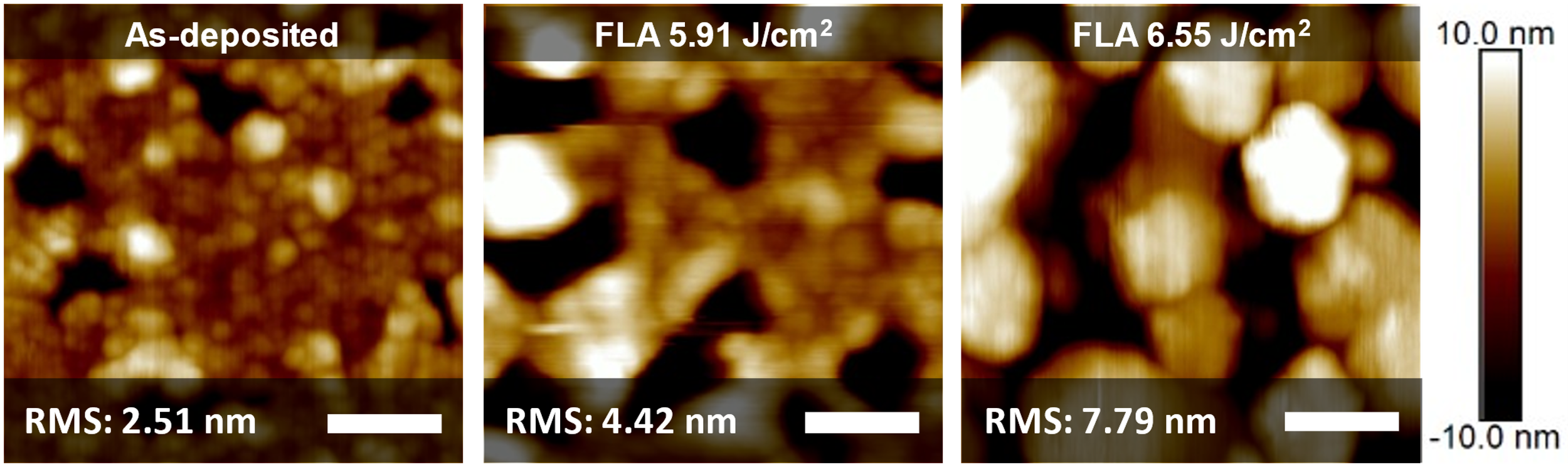


Supplementary Figure 8. Atomic force microscopy (AFM) images of as-deposited and flash lamp annealed Ag single layer films. The scale bar is 100 nm.


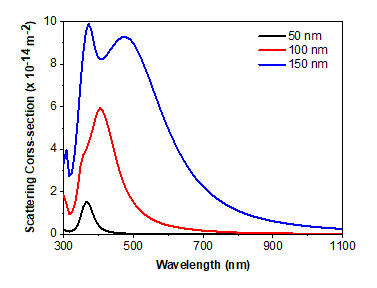


Supplementary Figure 9. Calculated extinction cross-section spectra of spherical Ag nanoparticles for different diameters based on Mie theory.
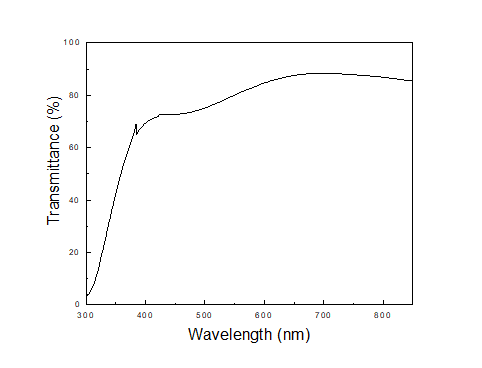


**Supplementary Figure 10.** Transmittance spectrum of IZO/Ag/IZO multilayer film which was thermally annealed at 500 ^o^C through traditional furnace heating.


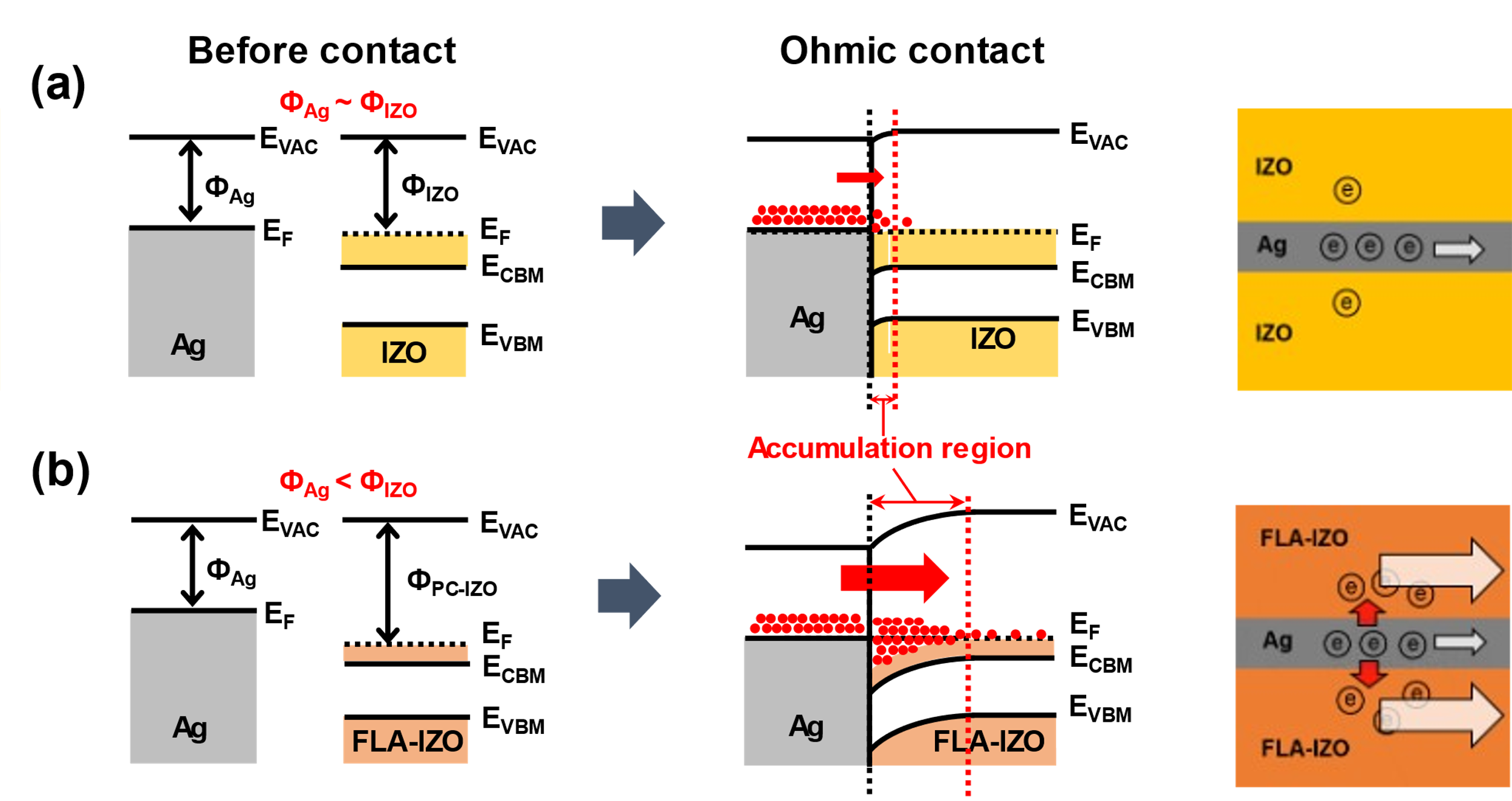


Supplementary Figure 11. Schematic illustration of ohmic contact formation between (a) Ag and IZO, and (b) Ag and flash lamp annealed IZO (FLA-IZO).


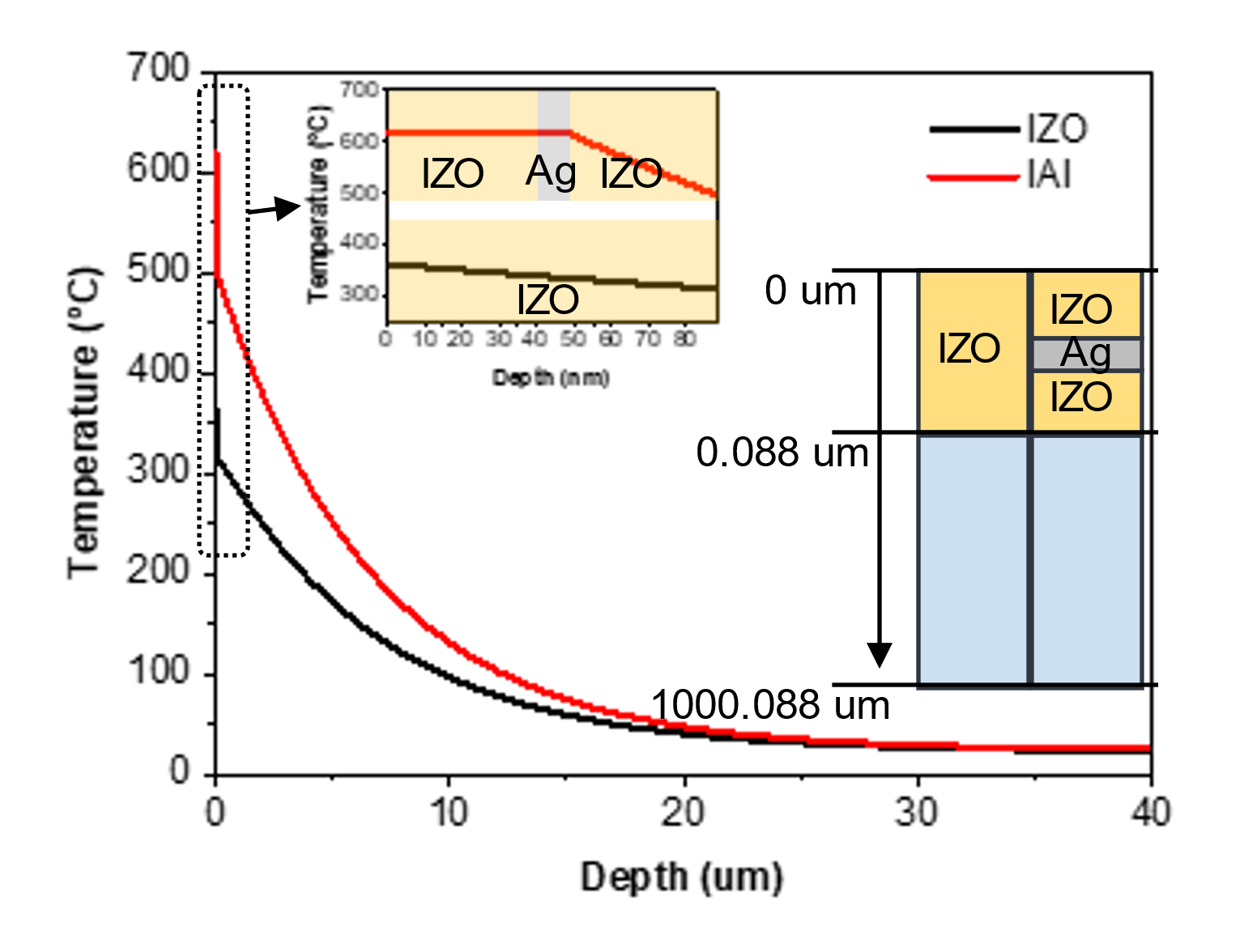


Supplementary Figure 12. Simulated temperature profile of IZO and IZO/Ag/IZO for depth direction at 0.2 ms of pulse duration (Pulse condition: 4.95 J/cm^2^ for 0.2 ms).


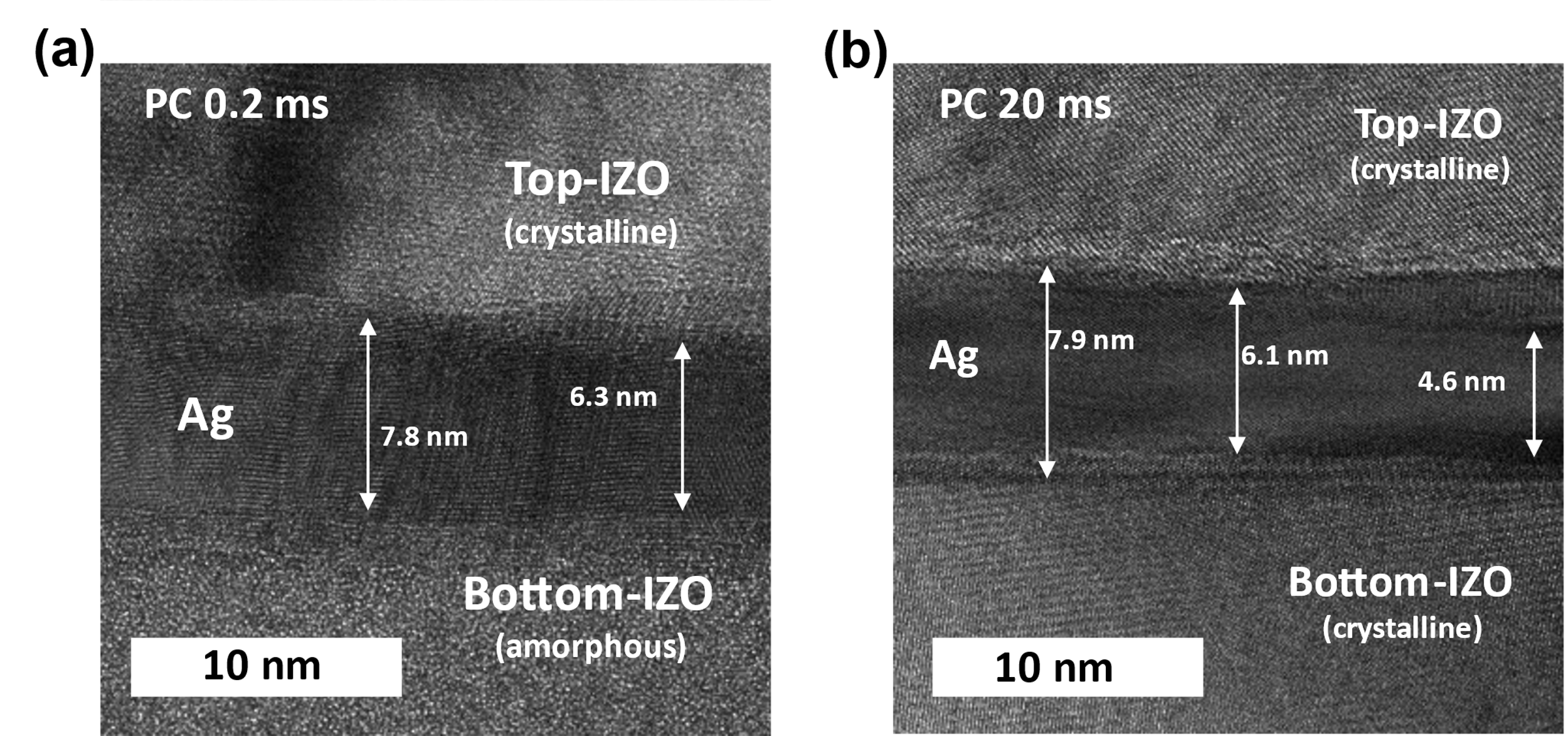


Supplementary Figure 13. Cross-sectional HR-TEM images of flash lamp annealed IZO/Ag/IZO multilayer film for (a) 0.2 ms and (b) 20 ms of pulse durations with the fixed peak temperature (620 °C).


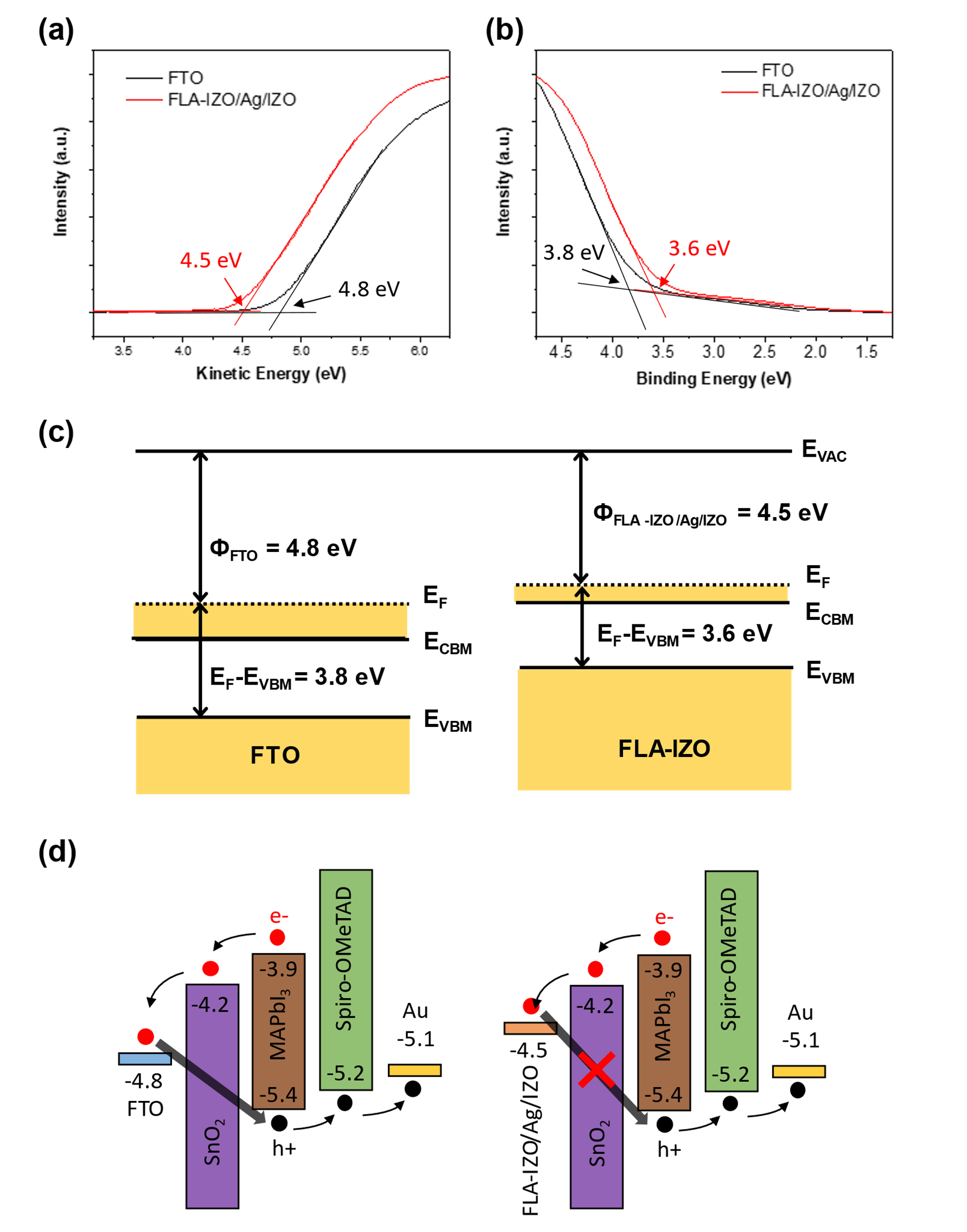


Supplementary Figure 14. (a) Secondary electron cut-off and (b) valance spectrum of the FTO and FLA-IZO/Ag/IZO measured with UPS. (c) Schematic band diagram of FTO and FLA-IZO based on the UPS. (d) Energy level diagram of perovskite solar cells with FTO and FLA-IZO/Ag/IZO.


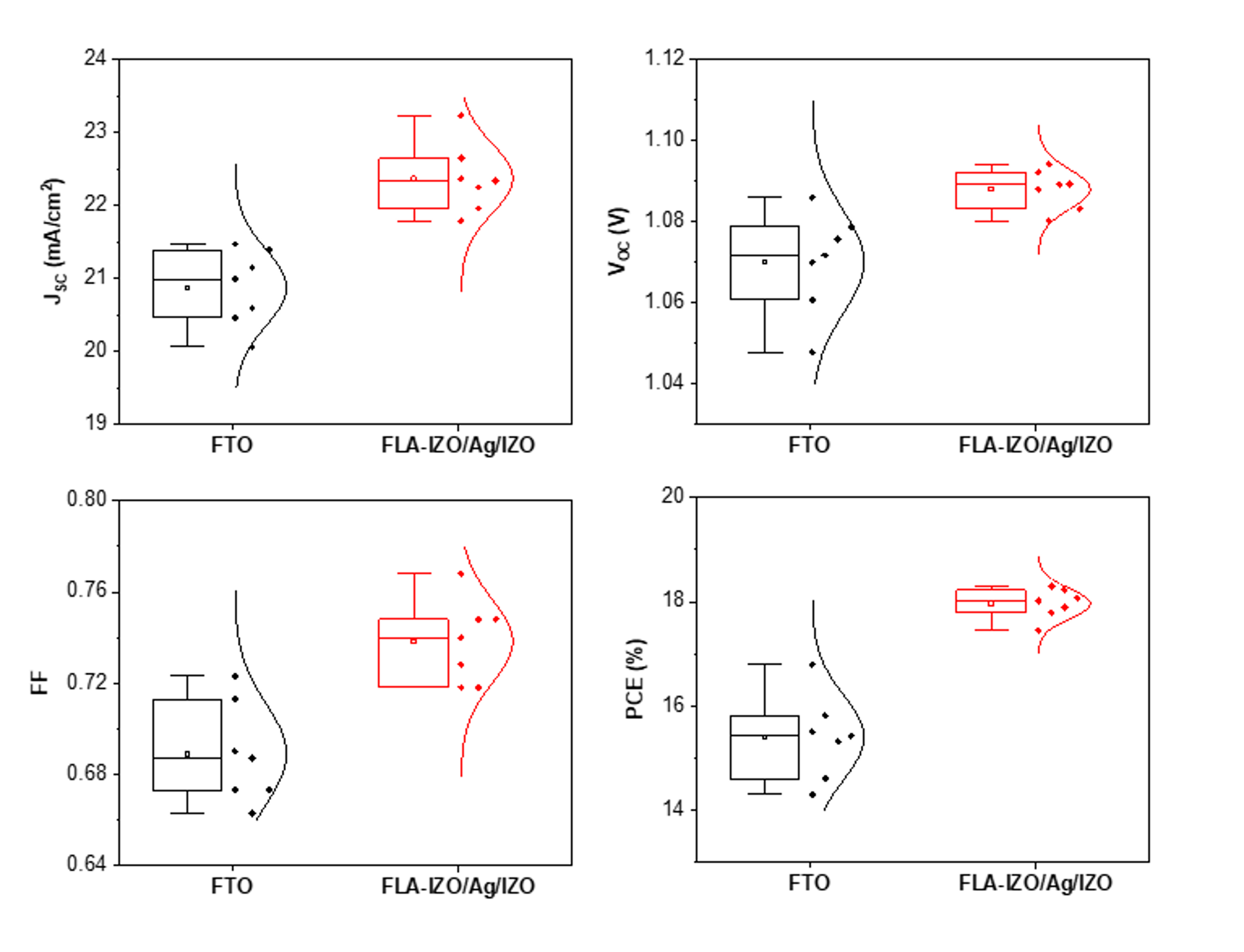


Supplementary Figure 15. Statistical distributions of the J_SC_, V_OC_, FF and PCE of solar cell devices with FTO (black) and FLA-IZO/Ag/IZO (red) electrodes.
